# Molecular Characterization of Natural Hybrids Formed between Five Related Indigenous Clade 6 *Phytophthora* Species

**DOI:** 10.1371/journal.pone.0134225

**Published:** 2015-08-06

**Authors:** Treena I. Burgess

**Affiliations:** Centre for Phytophthora Science and Management, School of Veterinary and Life Sciences, Murdoch University, 90 South Street, Murdoch, WA, 6150, Australia; Agriculture and Agri-Food Canada, CANADA

## Abstract

Most *Phytophthora* hybrids characterized to date have emerged from nurseries and managed landscapes, most likely generated as a consequence of biological invasions associated with the movement of living plants and germplasm for ornamental, horticultural and agricultural purposes. Presented here is evidence for natural hybridization among a group of five closely related indigenous clade 6 *Phytophthora* species isolated from waterways and riparian ecosystems in Western Australia. Molecular characterization of hybrids consisted of cloning and sequencing two nuclear genes (ITS and ASF), sequencing of two further nuclear loci (BT and HSP) and of two mitochondrial loci (COI and NADH). Additionally, phenotypic traits including morphology of sporangia and optima and maxima temperatures for growth were also determined. In most cases the nuclear genes were biparentally and in all cases the mtDNA were uniparentally inherited, indicating hybrid formation through sexual crosses. Some isolates bear the molecular signature of three parents suggesting additional hybrid events, although it cannot be determined from the data if these were sequential or simultaneous. These species and their hybrids co-exist in riparian ecosystems and waterways where their ability for rapid asexual proliferation would enable them to rapidly colonize green plant litter. The apparent ease of hybridization could eventually lead to the merging of species through introgression. However, at this point in time, species integrity has been maintained and a more likely scenario is that the hybrids are not stable evolutionary lineages, but rather transient hybrid clones.

## Introduction

*Phytophthora* and other Oomycetes produce hyphal systems and have similar life strategies to true fungi, however taxonomically they distantly related, with the true fungi residing in the Opisthokonts the Oomycetes in the Heterokontophyta [1]. Over the past decade it has become apparent that intraspecific hybridisation is a common occurrence in the genus *Phytophthora* and as such provides these plant pathogens with huge genomic plasticity. What is not yet clear is whether these hybridisation events would have occurred without anthropogenic activities reuniting closely related allopatric species [2]. During sympatric speciation reproductive incompatibility must develop to effectively maintain species integrity and avoid species fusion [3–5], however, closely related allopatric species do not have the same selection pressure to develop reproductive barriers. Greater pre-zygotic isolation in sympatry rather than allopatry is one of the main signatures of reinforcement, as has been empirically demonstrated for the brown macroalgal genus *Fucus* [6]. In four contact zones spanning 100–10,000 years, hybridisation and introgression between two *Fucus* spp. decreased with the increasing duration of sympatry. The phrase ‘happy rendezvous of old relatives’ has been coined by to describe hybridisation between related geographically isolated species when they are reunited by anthropogenic activity [7].

In all current phylogenies of *Phytophthora*, 10 strongly supported clades are recognised [8–10]. Reports of natural intraspecific hybridisation between related species in the genus *Phytophthora* have been on the rise in recent years and in all cases the hybrids form between species from the same phylogenetic clade. The most notorious example is *P*. x *alni*, a clade 7 hybrid thought to have arisen in tree nurseries before its introduction to the natural environment where it caused a new disease of alder in Europe [11–13]. Also well known are clade 1 hybrids between *P*. *nicotianae* and *P*. *cactorum*, now known as *P*. *x pelgrandis*, and hybrids between *P*. *cactorum* and *P*. *hedraiandra*, described as *P*. x *serendipita*, which have both been associated with disease in nurseries and horticulture [14–17]. Also in clade 1 is *P*. *andina*, a hybrid between *P*. *infestans* and a related but unknown species pathogenic toward fruits and foliage of wild and cultivated *Solanum* spp. [18]. Recently, three hybrids were described from clade 8b; all are pathogens of winter grown vegetables with different hosts preferences when compared to the parental species, *P*. *porri*, *P*. *primulae* and *P*. taxon parsley [19]. Several hybrids have been described from clade 6 including four between *P*. *chlamydospora* (a common resident of northern hemisphere streams) and *P*. *amnicola* and *P*. *thermophila* (southern hemisphere species) recovered from natural waterways in Australian and South Africa [20]. Also from clade 6 is *P*. *x stagnum*, a hybrid between *P*. *chlamydospora* and *P*. *mississippiae* recovered from nursery irrigation water [21]. A putative clade 9 hybrid isolates have also been recovered from waterways in South Africa [22].

Several of the described hybrids are apparently sterile (or at least sterile under laboratory conditions) or have reduced reproductive success probably due to meiotic incompatibility, often observed as abortive, unviable or deformed oospores and poor germtube development [11,19,21,23,24]. Among hybrids for which chromosome number has been determined, many exhibit polyploidy [12,19]. Polyploidy *per se* is common in the genus *Phytophthora* [25] with evidence for historical whole genome duplication [26,27]

Most of these new hybrid taxa are apparently a by-products of globalisation, with human activities associated with nurseries, ornamental gardens, horticulture and agriculture [2,28,29]. These are environments where infected germplasm and other material (such as soil) from disparate origins are bought together, providing a natural laboratory for the formation and selection of hybrids [7,30,31]. It is not surprising that hybrids form between closely related species as they share common ancestors and, if they have evolved through geographic isolation (allopatric speciation), they may have no intrinsic reproductive boundaries [5–7,32], especially if the species have same chromosome number which would allow for homoploid hybridisation [33]. However, while there is evidence to support hybridisation between reunited sister taxa in *Phytophthora*, there are few studies about naturally forming hybrids, between indigenous, related sympatric species. Natural hybridisation has been shown for reed associated *Pythium* species in Europe [34].

In the past few years five closely related but distinct *Phytophthora* species have been described from natural waterways in the south-west of Western Australia (WA); *P*. *thermophila* and *P*. *litoralis* [35], *P*. *fluvialis* [36], *P*. *amnicola* [37] and *P*. *moyootj* [38]. These species are thought to be indigenous to the region and fill the ecological niche that related clade 6 species, *P*. *lacustris*, *P*. *gonapodyides* and *P*. *chlamydospora*, are known to fill in Europe and Northern America [39–42]. In the same routine surveys in which the aforementioned species were recovered, numerous isolates that either exhibited additivity in ITS region or produced mixed unreadable sequence were also isolated [43]. Molecular and phenotypic characterisation of representative isolates is presented here.

## Materials and Methods

### Ethics Statement

The putative hybrid characterized in this study (Table 1) were recovered during routine sampling by the Vegetation Health Service (VHS) of the Department of Parks and Wildlife, Western Australia or during the Fishing for *Phytophthora* Project [43]. Local government authorities, Natural Resource Management (NRM) groups and dieback interpreters collected the isolates during routine surveys for which no specific permissions were required. These activities did not involve endangered or protected species.

**Table 1:** *Phytophthora* isolates considered in this study.

Species^a^	Code^b^	Isolate	Substrate	Source	Location	Collector	Year
*Phytophthora amnicola*	A	CBS131652	Water	stream baiting	Lake Jualbup, WA, Australia	D Hüberli	2009
*P*. *amnicola*	A	VHS19503	Soil	*Patersonia* sp.	Pemberton, WA, Australia	VHS	2008
*P*. *amnicola x P*. *moyootj*	A-M	DH180	water	stream baiting	Australia, WA, MGS-Kotisinia	D Hüberli	2009
*P*. *amnicola x P*. *moyootj*	A-M	DH283	water	stream baiting	Australia, WA, Bibra lake	D Hüberli	2009
*P*. *amnicola x P*. *moyootj*	A-M	DH284	water	stream baiting	Australia, WA, Bibra lake	D Hüberli	2009
*P*. *asparagi*		VHS17175	Soil	*Banksia media*	Esperance, WA, Australia	VHS	2007
*P*. *fluvialis*	F	CBS129424	Water	stream baiting	Moore River, WA, Australia	D Hüberli	2009
*P*. *fluvialis*	F	VHS17350	Water	stream baiting	Badgingarra, WA, Australia	VHS	2007
*P*. *fluvialis x P*. *moyootj*	F-M	DH011	water	stream baiting	Australia, WA, Gnowangerup Creek	D Hüberli	2009
*P*. *fluvialis x P*. *moyootj*	F-M	DH087	water	stream baiting	Australia, WA, Howatharra Road Creek	D Hüberli	2009
*P*. *fluvialis x P*. *moyootj*	F-M	DH089	water	stream baiting	Australia, WA, Chapman River	D Hüberli	2009
*P*. *fluvialis x P*. *moyootj*	F-M	DDS3642	water	native vegetation	Australia, WA, Albany	VHS	1994
*P*. *fluvialis* hybrid		DH117	water	stream baiting	Australia, WA, Wooroloo	D Hüberli	2009
*P*. *fluvialis* hybrid		VHS29992	Soil	*Banksia grandis*	Australia, WA, Boddington	VHS	2013
*P*. *fragariae*		CBS309.62	Plant	*Fragaria × ananassa*	Scotland, United Kingdom	CJ Hickman	1962
*P*. *gonapodyides*		MUCC776	Water	stream baiting	Australia, TAS	Y Ziqing	2009
*P*. *humicola*		P6702	Plant	*Phaseolus* sp.	Taiwan	P Ann	
*P*. *inundata*		VHS16836	Soil	*Xanthorrea*. *preissii*	Australia, WA, Boyup Brook	VHS	2007
*P*. *lacustris*		HSA1959	Soil	roadside drainage	Australia, WA, Welshpool	R Hart	1994
*P*. *litoralis*	L	VHS17085	Soil	*Banksia* sp.	Hopetoun, WA, Australia	VHS	2007
*P*. *litoralis*	L	CBS127953	Soil	*Banksia* sp.	Ravensthorpe, WA, Australia	VHS	2008
*P*. *litoralis x P*. *moyootj*	L-M	DH134	water	stream baiting	Australia, Stockyards Creek	D Hüberli	2009
*P*. *litoralis x P*. *moyootj*	L-M	DH147	water	stream baiting	Australia, WA, Young River	D Hüberli	2009
*P*. *litoralis x P*. *moyootj*	L-M	VHS16115	Soil	native vegetation	Australia, WA, Fitzgerald River NP	VHS	2006
*P*. *megasperma*		DDS3432	Soil	*Banksia* sp.	North Dinninup, WA, Australia	VHS	1992
*P*. *mooyotj*	M	VHS16108	Soil	native vegetation	Australia, WA, Fitzgerald River NP	VHS	2006
*P*. *mooyotj*	M	VHS27218	Soil	mud	Australia, WA, Walpole	VHS	2012
*P*. *mooyotj*	M	DH103	Soil	restored minepit	Australia, WA, Jarrahdale	D Hüberli	2012
*P*. *moyootj*	M	DH056	water	stream baiting	Australia, WA, Steere River	D Hüberli	2009
*P*. *moyootj*	M	DH137	water	stream baiting	Australia, WA, Coramup Creek Reserve	D Hüberli	2009
*P*. *moyootj*	M	DH206	water	stream baiting	Australia, WA, Jerdacuttup River	D Hüberli	2009
*P*. *moyootj x P*. *fluvialis*	M-F	BAN-A	Water	stream baiting	Australia, WA, Banister River	T Burgess	2011
*P*. *moyootj x P*. *fluvialis*	M-F	DH181	water	stream baiting	Australia, WA, Lake Coogee	D Hüberli	2009
*P*. *moyootj x P*. *fluvialis*	M-F	DH182	water	stream baiting	Australia, WA, Lake Coogee	D Hüberli	2009
*P*. *moyootj x P*. *fluvialis*	M-F	DH286	water	stream baiting	Australia, WA, Lake Coogee	D Hüberli	2009
*P*. *moyootj x P*. *litoralis*	M-F	MUR-C	water	stream baiting	Australia, WA, Murray River	T Burgess	2011
*P*. *moyootj x P*. *thermophila*	M-T	BAN-B	water	stream baiting	Australia, WA, Banister River	T Burgess	2011
*P*. *moyootj x P*. *thermophila*	M-T	MUR-A	water	stream baiting	Australia, WA, Murray River	T Burgess	2011
*P*. *sp*.*nov*. hybrid		VHS2713	Soil	native vegetation	Australia, WA, Deep River	VHS	1997
*P*. *sp*. *nov x P*.*amnicola*	U-A	DH269	water	stream baiting	Australia, WA, Upper Denmark River	D Hüberli	2009
*P*. *chlamydospora*		VHS6595	Soil	Native forest	Manjimup, WA, Australia	VHS	1999
*P*. *thermophila*	T	VHS7474	Soil	Native forest	Manjimup, WA, Australia	VHS	2000
*P*. *thermophila*	T	VHS13530	Soil	*Eucalyptus marginata*	Dwellingup, WA, Australia	VHS	2004
*P*. *thermophila* hybrid		DH106	water	stream baiting	Australia, WA, Jarrahdale	D Hüberli	2009
*P*. *thermophila x P*. *amnicola*	T-A	DH150	water	stream baiting	Australia, WA, Lake Jualbup	D Hüberli	2009
*P*. *thermophila x P*. *amnicola*	T-A	VHS5185	Soil	native vegetation	Australia, WA, Pemberton	VHS	1998
*P*. *thermophila x P*. *amnicola*	T-A	VHS22715	Soil	urban parkland	Australia, WA, Perth, Mosman Park	VHS	2009
*P*. *thermophila x P*.*moyootj*	T-M	DH265	water	stream baiting	Australia, WA, Upper Hay River River	D Hüberli	2009

^a^ Hybrid names are given as maternal x paternal parent

^b^ abbreviated names as used throughout the manuscript.

U = unknown species known only from *cox*I and NADH alleles

### Isolate maintenance

Isolates are maintained in the Murdoch University Culture Collection and the Vegetation Health Service Collection, Department of Parks and Wildlife, Western Australia. Included for comparative purposes were the parental species and three isolates collected from Western Australia, VHS5185, DH180 and VHS22715 previously characterized in the study of Nagel et al. [20] (Table 1). These isolates had been obtained in several ways; VHS isolates are from direct baiting of soil from beneath dead or dying vegetation. DH isolates were obtained through direct plating of leaves that had been suspended in streams. All other isolates were obtained by filtering of water and placing the filter onto *Phytophthora* selective media.

### DNA extraction, PCR, cloning and sequencing

Genomic DNA was extracted from isolates as described previously [44]. The region spanning the ITS1-5.8S-ITS2 region of the ribosomal DNA was usually amplified using the primers DC6 [8] and ITS-4 [45] however, if the amplicons were to be cloned then ITS6 [45] was used instead of DC6. The PCR reaction mixture and PCR conditions were as described previously [44]. For products that were to be cloned, GoTaq Hot Start Polymerase (Promega, Madison, USA) and buffer were used. The mitochondrial gene *cox*I (COI) and the anti-silencing factor (ASF)-like gene were amplified as described previously [20]. Heat shock protein 90 (HSP) was amplified with HSP90-F1 and HSP90-R2 [46] and the product was sequenced in both directions with primers HSP90-F1int and HSP90-R1; the PCR reaction mixture was the same as for the ITS region, but the PCR conditions were as described previously [46]. β-tubulin (BT) was amplified with primers TUBU-F2 and TUBU-R1 and NADH dehydrogenase subunit 1 was amplified and sequenced with NADH-F1 and NADH-R1 as described previously [9]. The ITS and ASF-like amplicons were cloned into a bacterial plasmid vector, pGEM-T Easy Vector System as described previously [20]. For ITS region, 10–80 amplicons were sequenced for each of the putative hybrids, while for ASF-like gene 6–8 amplicons were sequenced. The cleanup of amplicons using sephadex and sequencing were as described previously [47]. All sequences derived in this study were deposited in GenBank and accession numbers are given in S1 Table.

### Analysis of polymorphisms in ITS sequence data

**Fig 1:**
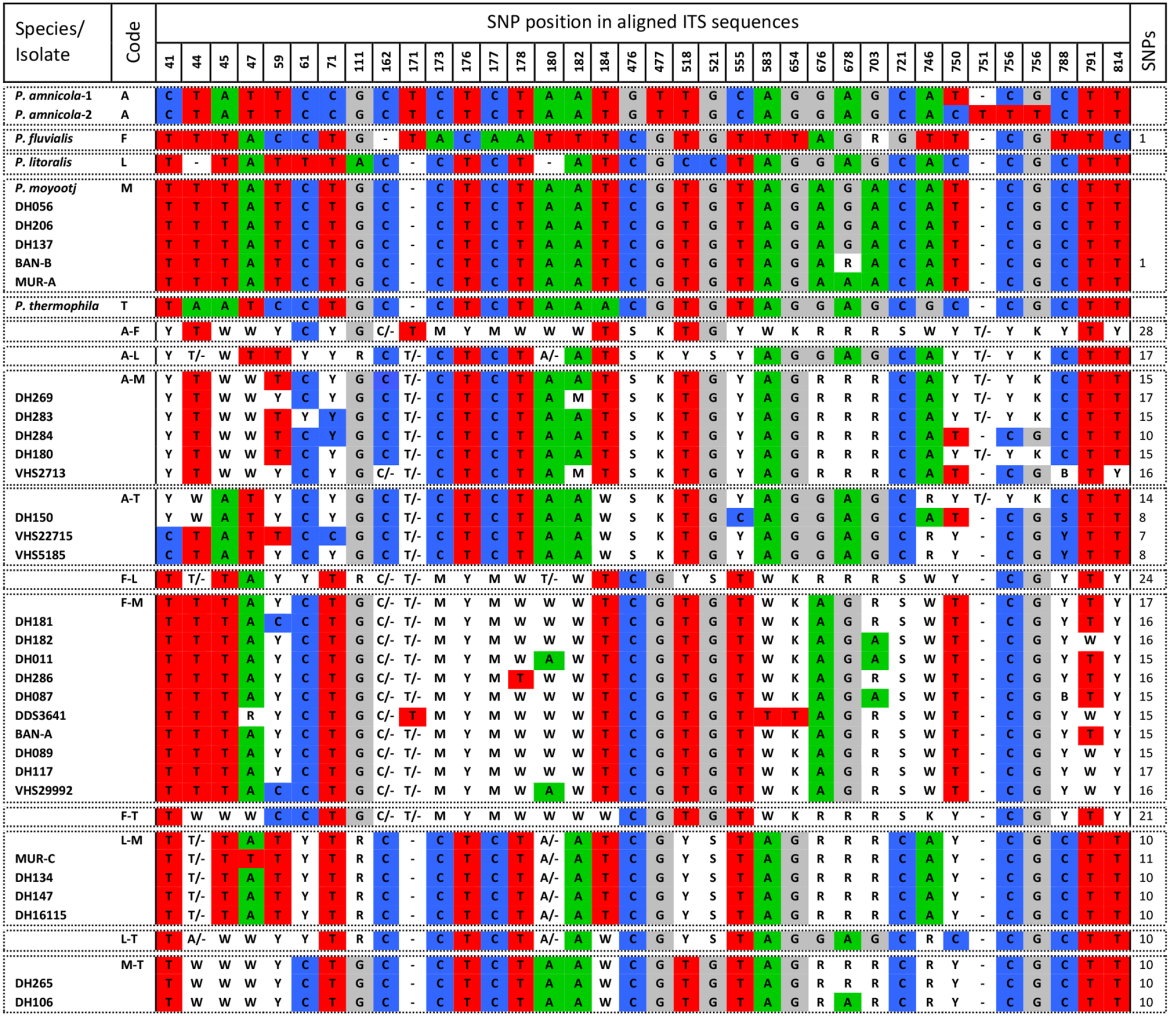
Polymorphisms in ITS sequence of *Phytophthora amnicola*, *P*. *fluvialis*, *P*. *litoralis*, *P*. *moyootj*, *P*. *thermophila* and related hybrids.

Data from the sequenced ITS amplicons of the putative hybrids suggested that five parental species were involved in producing the hybrids. These included *P*. *amnicola*, *P*. *fluvialis*, *P*. *litoralis*, *P*. *moyootj* and *P*. *thermophila*. For each hybrid isolate, the amplicons were aligned in Geneious 7.1.7 [48] and a consensus sequence was generated. Ambiguous bases were coded according to the IUPAC nucleotide code. Consensus sequences were also generated for each parental species from all available sequence data and the consensus sequences were then aligned Geneious 7.1.7 and interspecific single nucleotide polymorphisms (SNPs) identified. This alignment was used to construct the expected sequence of hybrid combinations between the parental species. There are 10 potential hybrid combinations between species designated as A-F, A-L, A-M, A-T, F-L, F-M, F-T, L-M, L-T and M-T (Table 2). The consensus sequences of the hybrid isolates were then aligned together with the parental species and the identity of the base at each of the identified positions were recorded (Fig 1).

**Table 2:** Identity of hybrid isolates based on mtDNA (*cox*I and NADH), three house keeping genes (ITS, HSP and BT) and the single copy ASF gene.

Isolate	*cox*I	NADH	ITS	HSP	BT	ASF
DH180	A	A	A-M	A-M	A-M	A-M
DH283	A	A	A-M	A	A-M	M
DH284	A	A	A-M	A	M	M
DDS3642	F	F	F-M	F-M	F-M	F-M
DH011	F	F	F-M	F-M	F-M	F-M
DH087	F	F	F-M	F-M	F-M	F-M
DH089	F	F	F-M	F	M	M
DH117	F	F	F-M	F-T	F-M	F-M
VHS29992	F	F	F-M	F-T	F-M	F-M
DH134	L	L	L-M	L-M	L-M	L-M
DH147	L	L	L-M	L-M	L-M	L-M
VHS16115	L	L	L-M	L-M	L-M	L-M
DH056^a^	M	M	M	M	M	M
DH137^a^	M	M	M	M	M	M
DH206^a^	M	M	M	M	M	M
DH269	U	U	A-M	A-M	M	A
VHS2713	U	U	A-M	A-F	A-F	A-F
BAN-A	M	M	M-F	M-F	F	M-F
DH181	M	M	M-F	M-F	M-F	M
DH182	M	M	M-F	M-F	M-F	M-F
DH286	M	M	M-F	M-F	M-F	M-F
MUR-C	M	M	M-L	M-L	M-L	M-L
BAN-B	M	M	M	M-T	M	M-T
MUR-A	M	M	M	T	M	M
DH150	T	T	T-A	T-A	T-A	T-A
VHS22715	T	T	T-A	T-A	T-A	T-A
VHS5185	T	T	T-A	T-A	T-A	T-A
DH265	T	T	T-M	T-M	T-M	T-M
DH106	T	T	T-M	F-M	T-M	T-F-M

^a^ based on current study these isolates are considered to represent a new genotype of *P*. *moyootj*

### Analysis of polymorphisms in HSP and BT sequence data

**Fig 2:**
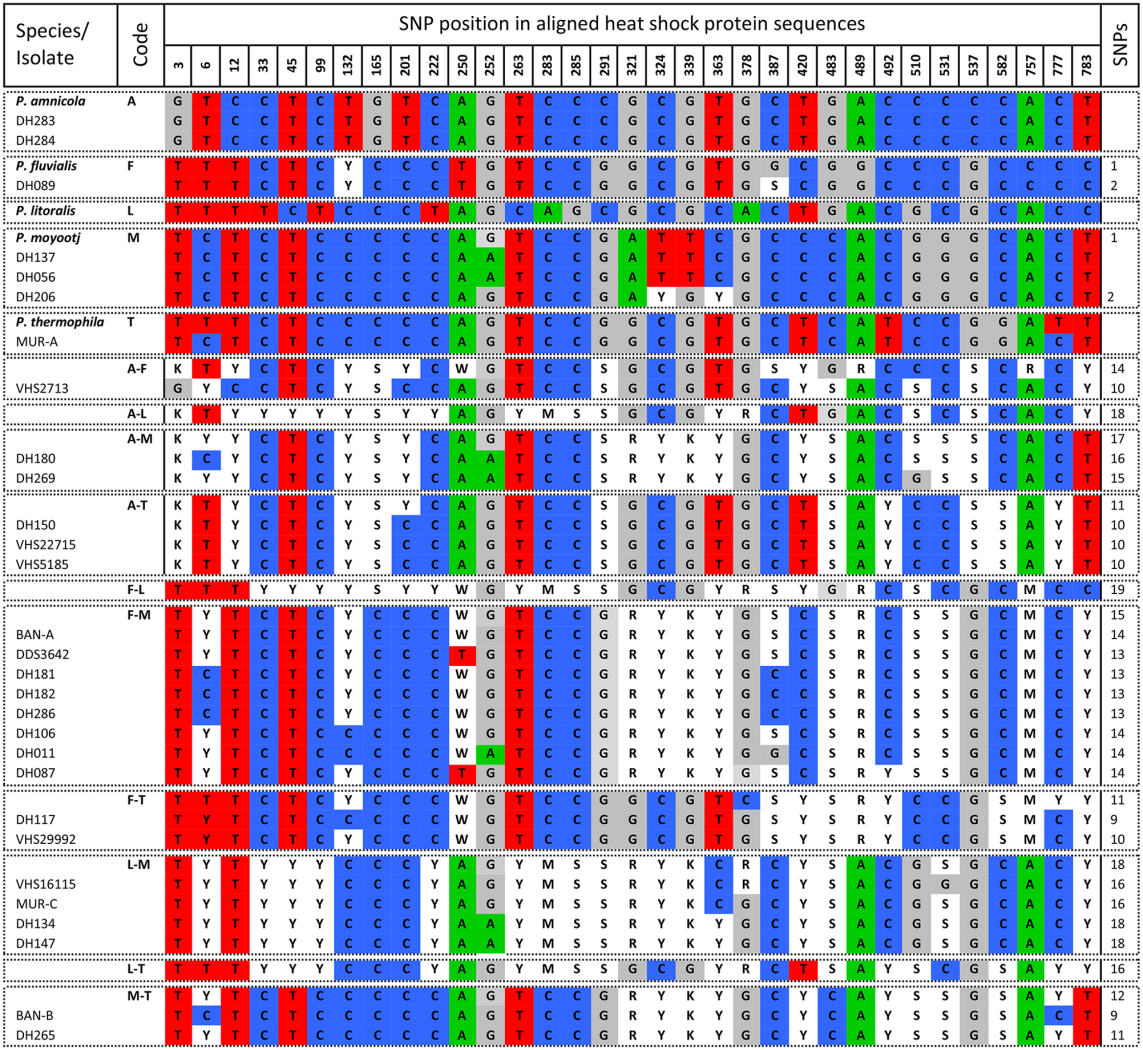
Polymorphisms in heat shock protein sequence of *Phytophthora amnicola*, *P*. *fluvialis*, *P*. *litoralis*, *P*. *moyootj*, *P*. *thermophila* and related hybrids.

**Fig 3:**
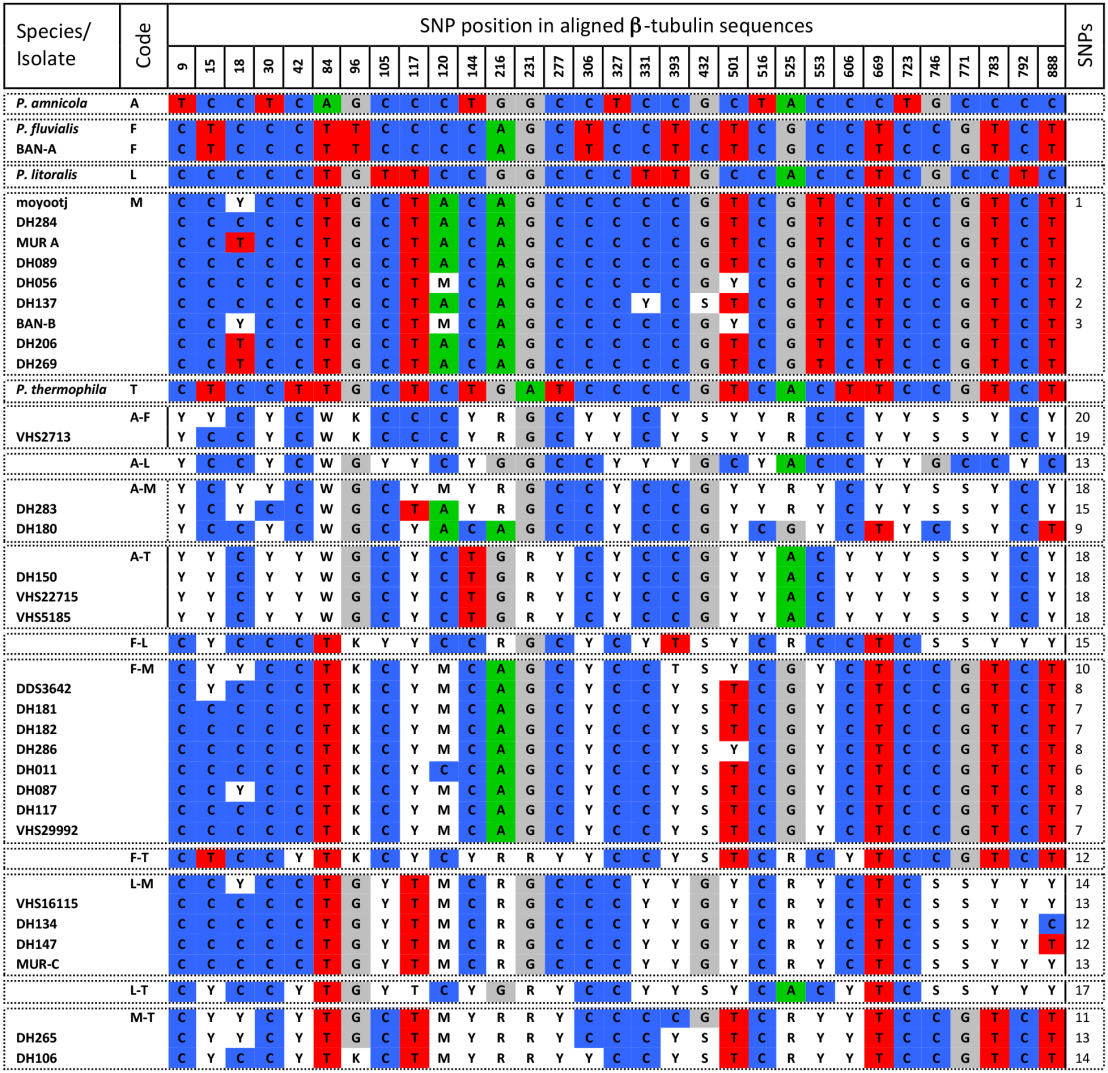
Polymorphisms in β-tubulin sequence of *Phytophthora amnicola*, *P*. *fluvialis*, *P*. *litoralis*, *P*. *moyootj*, *P*. *thermophila* and related hybrids.

Unlike the ITS sequence data which contains indels, the HSP and BT sequence is protein coding and contains no indels. As such, cloning of this region is unnecessary as SNPs can be identified as double peaks in the sequence chromatograms. Contigs were constructed from the forward and reverse sequences and consensus sequences generated for each hybrid isolate using IUPAC nucleotide code for SNPs. As for the ITS region, the consensus sequences of the parental species were aligned, interspecific SNPs identified and the expected sequences of the hybrid combinations were generated. The consensus sequences of the hybrid isolates were then aligned together with the parental species and the identity of the base at each of the identified SNPs was recorded (Figs 2 and 3).

### Phylogenetic analysis of ASF gene

Single copy nuclear genes, such as ASF, are excellent for identifying parental species of hybrids as they are, like rDNA, also biparentally inherited, however unlike rDNA they are not under concerted evolution and can be used to identify hybrids [13]. Sequence data were analyzed and edited in Geneious by combining forward and reverse sequences into contigs. Sequences representing each type of allele obtained for each isolate were retained for phylogenetic analysis. An alignment was generated in Geneious containing all parental species and the representative alleles from each hybrid isolate. Bayesian statistical inferences were used to generate phylogenetic trees and node support values. The optimal evolutionary model as determined using JModeltest [49] was the GTR+I+G model. Bayesian analyses were done using MrBayes Plugin [50] for Geneious and each analysis was run for 2 000 000 generations with burnin set at 10%. Alignment files and trees can be viewed on TreeBASE 16717 (http://www.treebase.org/).

### Phylogenetic analysis of mitochondrial genes

Mitochondrial genes are uniparentally inherited through the maternal line and can be used to determine the maternal parent of a sexually formed hybrid. Sequence data were analyzed and edited in Geneious and additional sequences were retrieved from GenBank (http://www.ncbi.nlm.nih.gov/). A concatenated alignment of COI and NADH was generated in Geneious containing all parental species and the representative alleles from each hybrid isolate and Bayesian analysis was conducted as described above using the GTR+G model.

### Morphological comparison

Many of the isolates identified through molecular studies to be hybrid isolates did not survive in standard water storage at 10°C for more than three months. Thus, morphological studies were conducted on a sub-set of isolates. Colony growth rates were determined for selected hybrid isolates, and compared to that of parental species. Plates, incubated at a high temperature and where no growth was observed, were subsequently moved to a 20°C incubator to establish their viability.

The dimensions of selected morphological characters were measured to further compare the hybrid groups with the five reference species; *P*. *amnicola* [37], *P*. *fluvialis* [36], *P*. *litoralis* [35], *P*. *moyootj* [38] and *P*. *thermophila* [35]. Sporangia, chlamydospores and hyphal swellings produced on V8A were measured using the methods described previously [35]. In order to stimulate the formation of gametangia, isolates were paired with *P*. *cinnamomi* tester strains and incubated at 20°C in the dark for 2–4 weeks.

## Results

### Analysis of polymorphisms in ITS sequence data

The alignment of the consensus sequences of the five reference *Phytophthora* species was 823 bp in length and SNP positions in all parental species and hybrids are based on this alignment (Fig 1). Two ITS alleles are present in some isolates of *P*. *amnicola* and both alleles have been included in the alignment; these alleles differ by a single 3 bp indel which prevents direct sequencing, but no other SNP’s have been found *P*. *amnicola* in any gene region [20]. Across the five parental species there are 36 fixed variable positions; 31 SNPs and five single bp indels (Fig 1) that for simplicity, shall all be referred to as SNPs. The number of SNPs between the different potential hybrid combinations is given in Fig 1.

The consensus ITS sequences obtained from hybrid isolates were added to the alignment of the five reference parental species (Fig 1). The identified interspecific SNPs were then used to compare the similarity of sequences from the hybrid isolates with the consensus sequences of the reference species and the expected sequences of the potential hybrid combinations. From this comparison, five hybrid groups could be identified, namely those with ITS sequences originating from a combination of *P*. *amnicola* and *P*. *moyootj* (DH180, DH269, DH283, DH284, VHS2713), *P*. *amnicola* and *P*. *thermophila* (VHS22715, VHS5185, DH150), *P*. *fluvialis* and *P*. *moyootj* (DH011, DH087, DH089, DH117, DH181, DH182, DH286, BAN-A, VHS3642, VHS29992), *P*. *litoralis* and *P*. *moyootj* (DH134, DH147, VHS16115, MUR-C) and *P*. *moyootj* and *P*. *thermophila* (DH106, DH265). Additionally five isolates had ITS sequence identical to that of *P*. *moyootj* (DH056, DH206, DH137, BAN-B, MUR-A). Combinations between *P*. *fluvialis* and *P*. *amnicola*, *P*. *litoralis* and *P*. *thermophila* were not observed, nor were combinations between *P*. *litoralis* and *P*. *amnicola* or *P*. *thermophila* (Fig 1).

S2 Table contains the frequency of all the ITS alleles resulting from cloning. For most hybrid isolates, the ITS haplotypes of the parents were recovered, but also recombinant ITS types. Exceptions were the F-M hybrid, DH089 and the T-M hybrid DH106 where only parental types were observed. Additionally for T-A hybrids, VHS5185, VHS22715 and DH150, the ITS types recovered were either identical to that of the *P*. *amnicola* parent or more similar to the *P*. *amnicola* parent than the *P*. *thermophila* parent, no ITS haplotypes of the *P*. *thermophila* parent were recovered (S2 Table).

### Analysis of polymorphisms in HSP and BT sequence data

The alignment of the consensus sequences of the five reference *Phytophthora* species for HSP was 798 bp in length and SNP position in all parental species and hybrids is based on this alignment (Fig 2). Across the five parental species there are 32 fixed SNPs with the number of SNPs between the different potential hybrid combinations is given in Fig 2. The consensus HSP sequences obtained from hybrid isolates were aligned with and compared to the five reference species and the expected sequences of all potential hybrid combinations and then compared (Fig 2). From the above comparison, seven hybrid groups could be identified; namely those with HSP sequences originating from a combination of *P*. *amnicola* and *P*. *fluvialis* (VHS2713), *P*. *amnicola* and *P*. *moyootj* (DH180, DH269) *P*. *amnicola* and *P*. *thermophila* (VHS22715, VHS5185, DH150), *P*. *fluvialis* and *P*. *moyootj* (DH011, DH087, DH089, DH181, DH182, DH286, BAN-A, VHS3642), *P*. *fluvialis* and *P*. *thermophila* (DH117, VHS29992), *P*. *litoralis* and *P*. *moyootj* (DH134, DH147, VHS16115, MUR-C) and *P*. *moyootj* and *P*. *thermophila* (BAN-B, DH265). Additionally two isolates had HSP sequence identical to that of *P*. *amnicola* (DH283, DH284), three were identical to *P*. *moyootj* (DH056, DH206, DH137), DH089 had sequence identical to *P*. *fluvialis* and MUR-A had sequence identical to *P*. *thermophila*. Combinations between *P*. *litoralis* and *P*. *amnicola*, *P*. *fluvialis* or *P*. *thermophila* were not observed (Fig 2).

The alignment of the consensus sequences of the five reference *Phytophthora* species for BT was 912 bp in length and SNP position in all parental species and hybrids is based on this alignment (Fig 3). Across the five parental species there are 31 fixed SNPs with the number of SNPs between the different potential hybrid combinations is given in Fig 3. The consensus HSP sequences obtained from hybrid isolates were aligned with and compared to the five reference species and the expected sequences of all potential hybrid combinations and then compared (Fig 3). From the above comparison, six hybrid groups could be identified; namely those with BT sequences originating from a combination of *P*. *amnicola* and *P*. *fluvialis* (VHS2713), *P*. *amnicola* and *P*. *moyootj* (DH180, DH283, DH269) *P*. *amnicola* and *P*. *thermophila* (VHS22715, VHS5185, DH150), *P*. *fluvialis* and *P*. *moyootj* (DH011, DH087, DH181, DH182, DH286, VHS3642, VHS29992), *P*. *litoralis* and *P*. *moyootj* (DH134, DH147, VHS16115, MUR-C) and *P*. *moyootj* and *P*. *thermophila* (DH106, DH265). Additionally two isolates had BT sequence identical to that of *P*. *moyootj* (DH056, DH206, DH137, DH284, MUR-A, DH089, BAN-B) and BAN-A had sequence identical to *P*. *fluvialis* Combinations between *P*. *fluvialis* and *P*. *thermophila* an between *P*. *litoralis* and *P*. *amnicola*, *P*. *fluvialis* or *P*. *thermophila* were not observed (Fig 3).

### Phylogenetic analysis of single copy ASF-like gene

**Fig 4:**
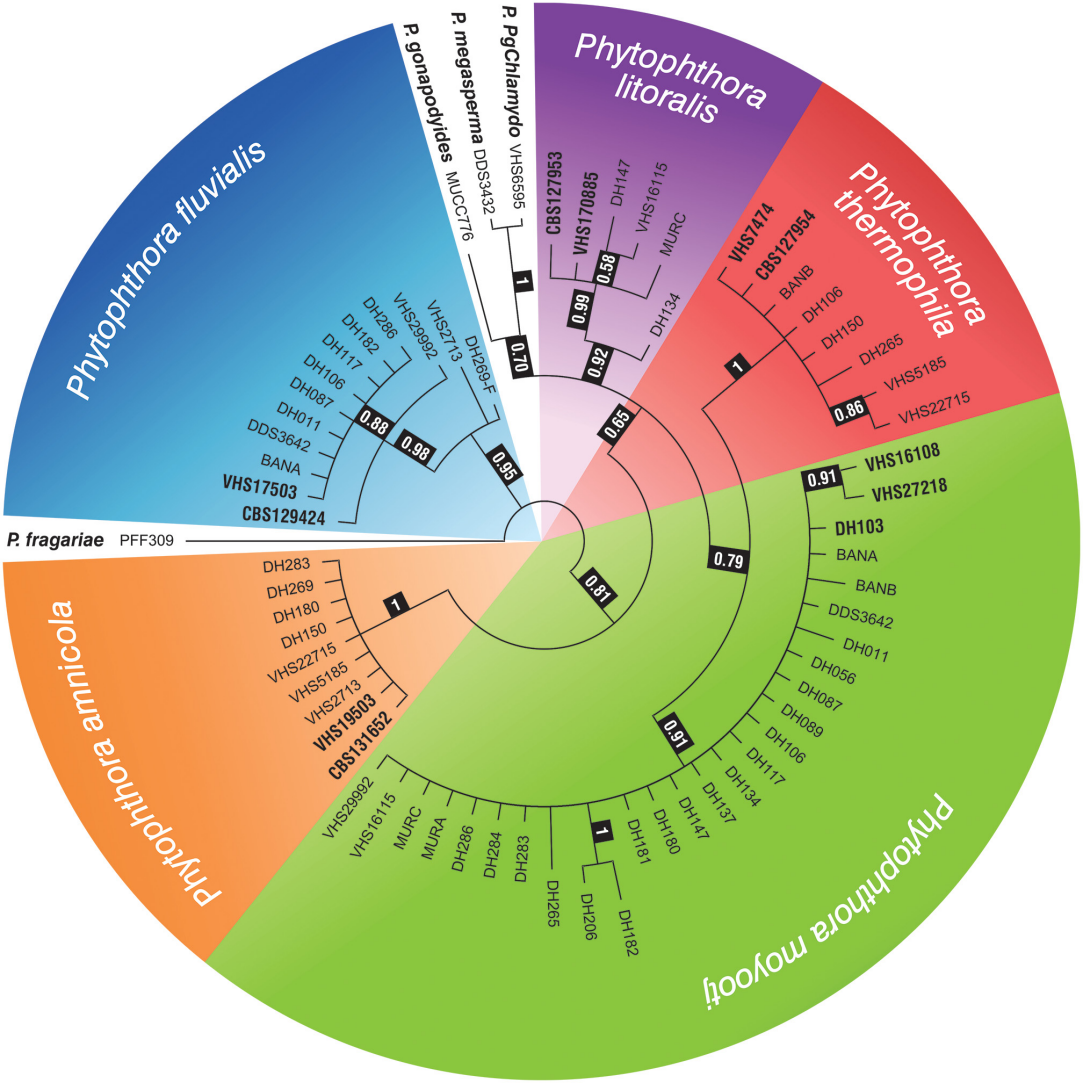
Bayesian inference tree based on ASF locus generated in MrBayes using the GTR +G substitution model. The posterior probability is shown at the nodes. Isolates of parental isolates are in bold. *Phytophthora fragariae* was used as an out-group taxon.

For each hybrid isolate, the amplicons were aligned and a consensus sequence was generated for each ASF haplotype (putative hybrid isolates had 1–3 ASF haplotypes). The alignment of the five parental species, all representative haplotypes identified from each hybrid isolate and additional reference species from *Phytophthora* clade 6 was 325 bp. *P*. *fragariae* was included as an out-group taxon. All haplotypes from all hybrid isolates resided in terminal clades corresponding to the five parental species (Fig 4). A summary of the ASF haplotypes observed for each isolate is given in Table 2.

### Phylogenetic relationships of mitochondrial genes

**Fig 5:**
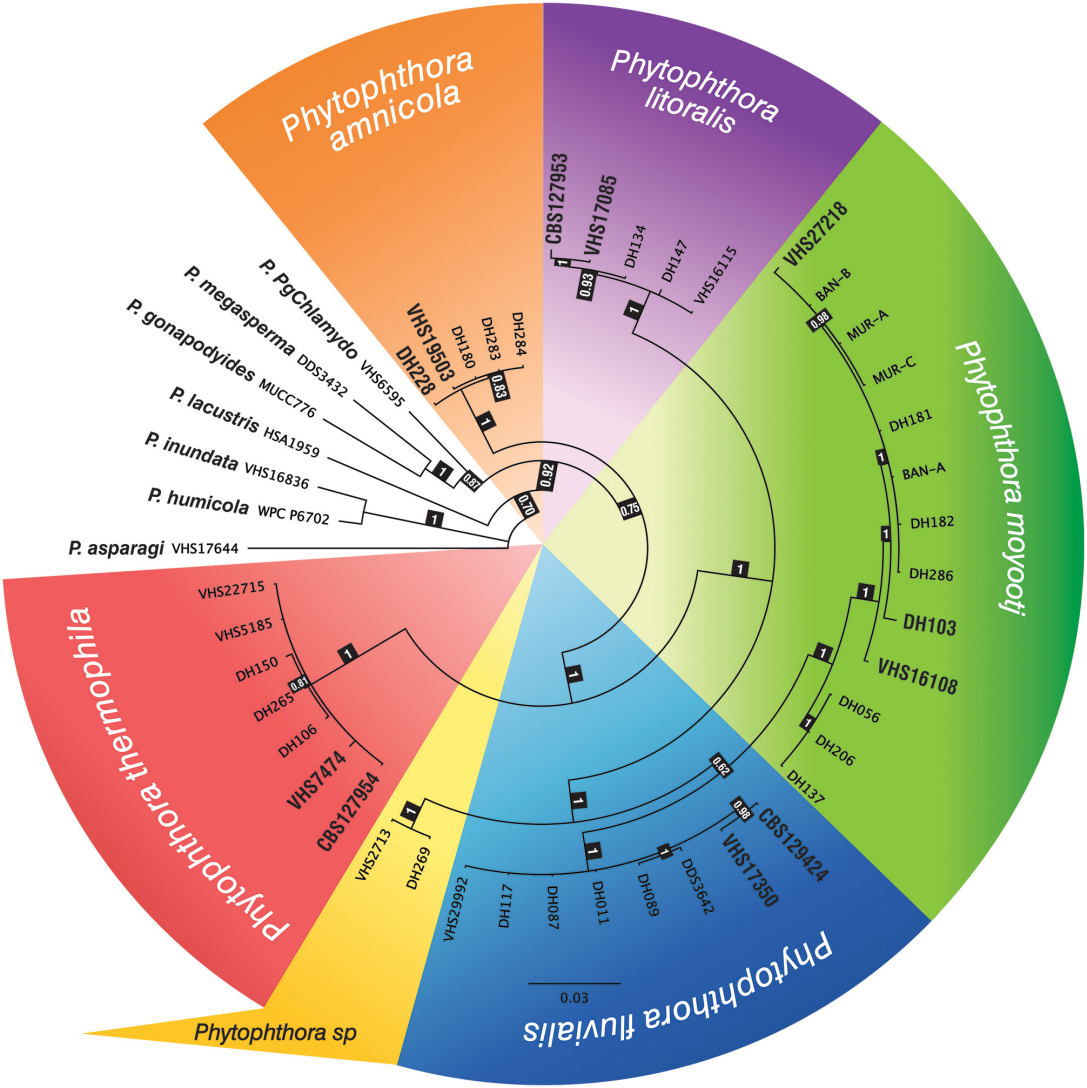
Bayesian inference tree based on concatenated mtDNA from COI and NADH loci generated in MrBayes using the GTR +G substitution model. The posterior probability is shown at the nodes. Isolates of parental isolates are in bold. *Phytophthora asparagi* was used as an out-group taxon.

**Fig 6:**
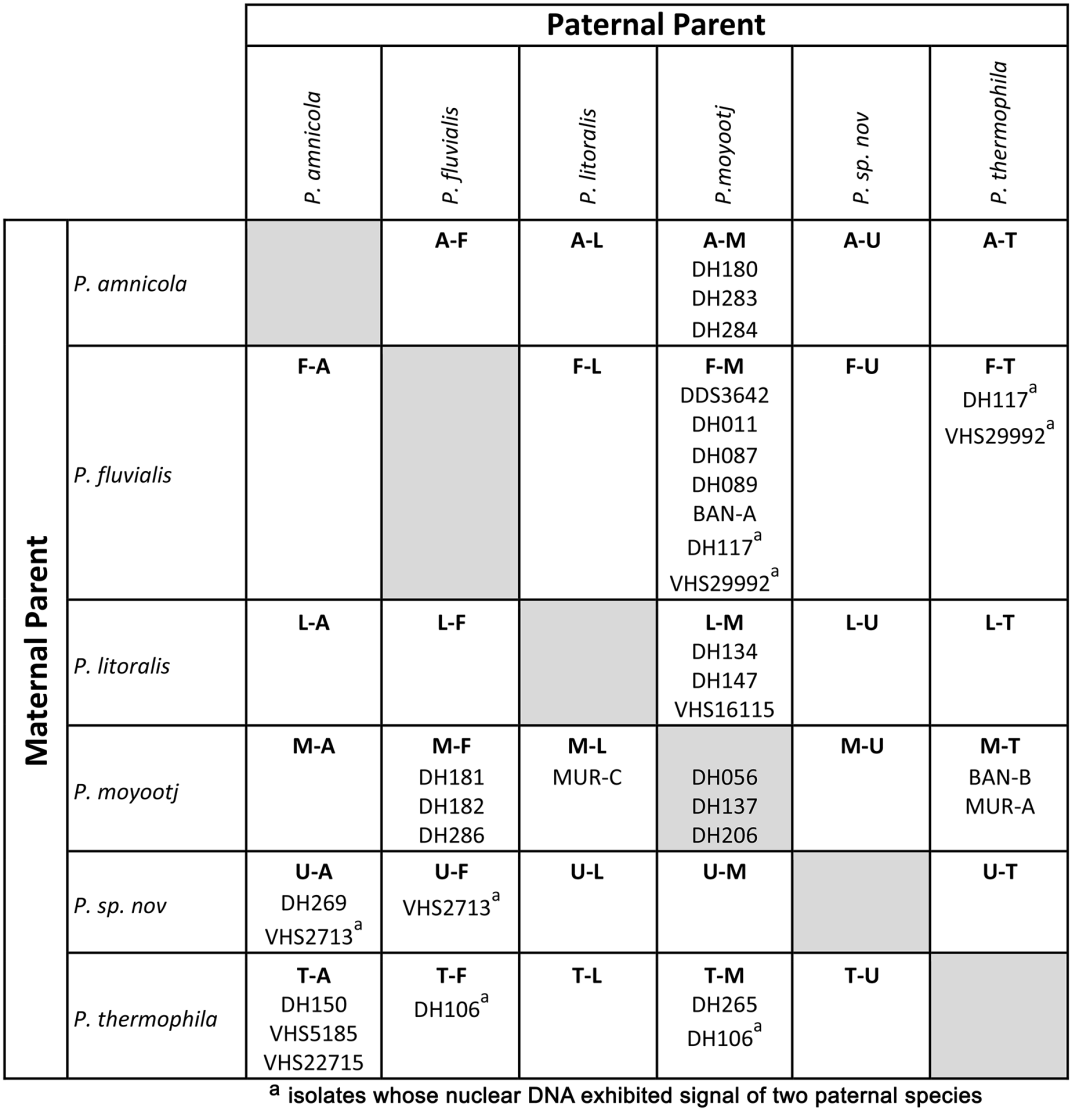
Placement of hybrid isolates into groups based on maternal (rows) and paternal (columns) parents. There are 30 possible hybrid combinations among the six potential parents.

COI and NADH were sequenced for all hybrid isolates and the concatenated alignment is 2009 bp. Most of the hybrid isolates clustered with one of the parental species (Fig 5). Isolates DH180, DH283 and DH284 resided with *P*. *amnicola*, isolates DH011, DH087, DH089, DH117, VHS29992 and DDS3642 resided with *P*. *fluvialis*, isolates DH134, DH147 and VHS16115 correspond to *P*. *litoralis*, DH181, DH182, DH286, MUR-C, BAN-A, BAN-B and MUR-A corresponded to *P*. *moyootj* while DH150, VHS22715, VHS5185, DH265 and DH106 resided with *P*. *thermophila*. Three isolates, DH056, DH137 and DH206 were identical to *P*. *moyootj* in all nuclear gene regions tested while their mtDNA clusters them together in a sister taxa to *P*. *moyootj* (Fig 5). These isolates are considered as representing natural intraspecific variation within *P*. *moyootj*. Two remaining isolates, VHS2713 and DH269, resided in their own clade in both in single gene and concatenated analyses of mtDNA. This clade does not correspond to any known species and could represent a sixth unknown species referred to as U (= unknown) in Table 2 and Fig 6.

### Paternity and hybridisation events among clade 6 species

The data from ITS, HSP and BT provide potential candidates for parents of this group of *Phytophthora* hybrids, which was confirmed by the sequencing of the single copy gene ASF. The mtDNA genes COI and NADH loci and by extension the mitochondrial genome was inherited uniparentally and allowed the maternal parent of the hybrid to be determined (Table 2). Among the six parents there are 30 possible hybrid combinations of which twelve were observed (Fig 6). Based on the International Code of Nomenclature for algae, fungi and plants (ICN) article H.2A.1; the name of the maternal parent precedes that of the male. Thus we propose the following terminology for the hybrids as follows: *P*. *amnicola x P*. *moyootj* (A-M), *P*. *fluvialis x P*. *moyootj* (F-M), *P*. *litoralis x P*. *moyootj* (L-M), *P*. *thermophila x P*. *moyootj* (T-M), *P*. *moyootj x P*. *fluvialis* (M-F), *P*. *moyootj x P*. *litoralis* (M-L), *P*. *moyootj x P*. *thermophila* (M-T) and *P*. *thermophila x P*. *amnicola* (T-A). Based on our data these simple two-way hybridizations account for 21 of the 26 hybrid isolates studied. The remaining 5 isolates produce a more complicated pattern.

Isolate DH206, appears based on nuclear genes, as a simple M-A hybrid, however the mtDNA alleles amplified do not match either potential parental species (VHS2713 has the same mtDNA profile). Isolates DH117 and VHS29992 have *P*. *fluvialis* as a maternal parent and contain ASF alleles for *P*. *fluvialis* and *P*. *moyootj* which should produce a simple F-M combination, however the SNP pattern observed in HSP was clearly that of a F-T hybrid. Isolate DH106 has *P*. *thermophila* as a maternal parent, however three ASF alleles were observed, *P*. *thermophila*, *P*. *moyootj* and *P*. *fluvialis*, ITS and BT sequences produced T-M profiles, but HSP produced an F-M profile. Isolate VHS2713 has the unknown mtDNA profile, ASF alleles corresponding to *P*. *amnicola* and *P*. *fluvialis* and HSP and BT SNP profiles corresponding to an A-F hybrid, but the ITS profile of an A-M hybrid. These five isolates with signals from more than two parents suggest they form through two hybridization events.

### Morphological comparisons

**Fig 7:**
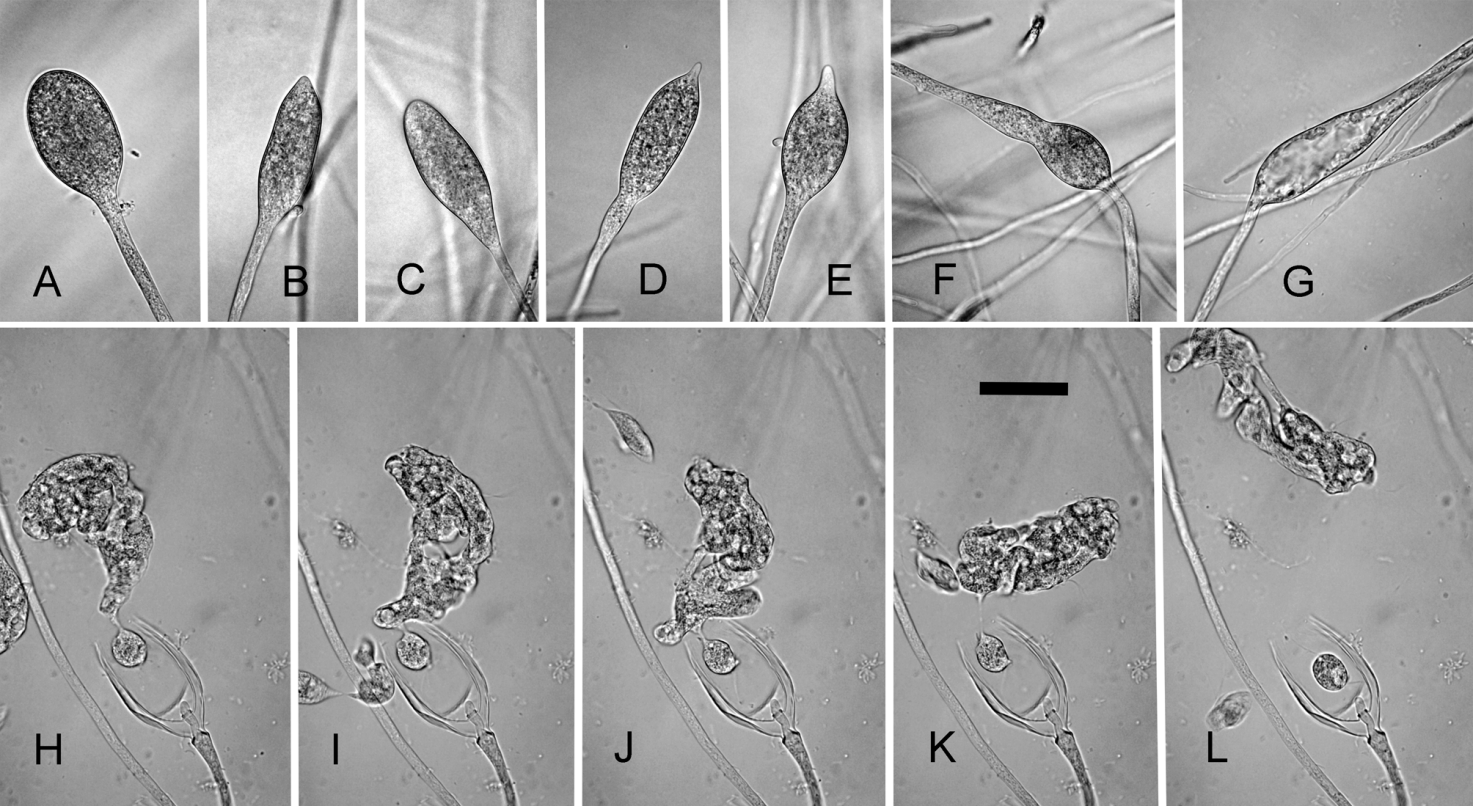
Morphological abnormalities observed in hybrid isolates. (A-G) DDS3642 and (H-L) DH180. (A-C) sporangial shapes with no septa (D-E) hyphae extending from apex (F-G) ‘sporangia’ reduced to swellings in hyphae. (H-L) time series of attempted zoospore release illustration the inability of the cytoplasm to cleave completely resulting in a clump of zoospores. Bar = 25 μm. Images: Thomas Jung

The five parental species have similar morphologies and life strategies (Table 3). They all produce abundant non-papillate, non-caducous sporangia predominantly ovoid in shape. Sporangia profusely proliferate internally, both nested and extended. External proliferation is also observed for *P*. *amnicola* and *P*. *litoralis*. Chlamydospores were rarely observed in *P*. *thermophila* and *P*. *litoralis* and hyphal swellings were observed for all species except *P*. *moyootj*. Most isolates of these species appear to be sterile in culture under laboratory conditions and would not form oogonia with tester strains, although one isolate of *P*. *thermophila* formed oospores in soil filtrate and one isolate of *P*. *litoralis* induce oogonia in an A2 tester strain [35]. Temperature optima for all species was greater than 30°C, with maximum temperature for growth on solid agar between 35 and 38°C depending on the species and the isolate. The majority of hybrid isolates measured had very similar morphology to the parental species; predominantly ovoid sporangia and profuse proliferation; their temperature optima were also similar (Table 3). Two isolates exhibited abnormal growth. Isolate DDS3642, a F-M hybrid, was unable for form sporangia; they started to develop (Fig 7A–7C), but septa failed to form and then the sporangia-like structure continued to grow from the apex (Fig 7D and 7E). Eventually the hyphae ended up with sporangia-like swellings (Fig 7F and 7G). Isolate DH180 was often unable to form zoospores, the cytoplasm in the sporangia cleaves and the zoospores are released, but they remain clumped, couldn’t separate and encysted as a blob (Fig 7H–7L).

**Table 3:** Summary of basic morphological features and growth of five parental species and hybrid isolates.

Isolate	Identity	Length	Breadth	L:B	Shapes^1^	Proliferation^2^	Chlamydo-spores	Optimal Temp. °C	Lethal Temp. °C
	*P*. *amnicola*	62.0±9.0	35.5±5.6	1.80	ov, lim	internal N and E and external	no	25–32.5	37.5
	*P*. *fluvialis*	53.0±7.7	36.4±6.1	1.50	ov, b-ov, lim	internal N and E and external	no	32	38.5
	*P*. *litoralis* ^*3*^	43.6±7.7	29.4±5.4	1.50	ov, el-ov, lim	internal N and E and external	yes	30	35
	*P*. *moyootj*	39.6±10.8	26.5±4.2	1.50	ov, b-ov, lim	internal N and E	no	32	35
	*P*. *thermophila* ^*4*^	44.8±6.3	25.7±3.9	1.80	ov, el-ov, lim	internal N and E	yes	33	37.5
VHS2713		41.5±4.0	33.2±2.8	1.25	ov	internal N and E	no	32.5	37.5
DH150	T-A	53.2±4.4	35.2±4.0	1.52	ov, lim, ell	internal N and E	no	30	37.5
VHS22715	T-A	46.0±5.8	29.9±4.9	1.56	ov, lim, ell	internal N and E	no	30	32.5
VHS5185	T-A	51.8±6.1	29.7±3.0	1.75	ov, lim, ell	internal N and E	no	30	35
DH180^5^	A-M	39.8±3.3	28.2±3.6	1.42	ov, ell, obp	internal N and E	no	30	32.5
DDS3642^6^	F-M	57.7±12.6	21.8±4.7	2.74	elongated		no	32.5	37.5
DH089	F-M	48.4±3.5	31.3±3.8	1.56	ov, ell	internal N and E	no	30	35
VHS16115	L-M	43.5±6.9	33.8±4.5	1.29	ov, ell, s-glob	internal N and E	no	32.5	<35

^1^ ov = ovoid, b-ov = broad ovoid, s-glob = sub-globose, el-ov = elongated ovoid, ell = ellipsoid, lim = limoniform,

^2^ N = nested, E = extended

^3^ Sterile or self sterile silent A1

^4^ Sterile or silent homothallic (1 isolate selfing in soil filtrate)

^5^ zoospores unable to cleave

^6^ sporangia unable to form

## Discussion

Hybridization between five related indigenous clade 6 *Phytophthora* species in Western Australian waterways appears to be a common phenomenon. At any given time, sampling (particularly from water) will yield a high proportion of hybrid isolates. In addition to the five described species an additional species was recognized in this study based solely on mtDNA. Out of the 30 potential hybrid combinations between these six parents, 12 were isolated and characterized here. *Phytophthora moyootj* was the species most commonly associated in hybrid combinations both as a maternal and paternal parent. Several of the isolates exhibited a clear DNA signal of three species. The hybrid isolates are all apparently sterile in culture and are very difficult to maintain in storage and may, in fact, be unstable transient entities.

### Hybrid characterisation

Without cloning, the ITS region is not a particularly useful locus for study interspecific hybrids as the presence of the highly variable non-coding regions in ITS1 and ITS2 and many indels leads not only to additivity, but also to unreadable sequence. Alternatively, other house keep genes such as BT and HSP are protein-coding regions and they also exhibit additivity, but this can easily be observed on the electropherograms and coded accordingly and, as such, there regions are well suited to this type of study. Ideally single copy nuclear genes, such as those studied for *P*. *alni* [13], are best suited for determining paternity. After testing a range of such genes, ASF was found to be the most variable and easiest to amplify for clade 6 *Phytophthora* spp. [20].

Maternal inheritance of mitochondria during sexual reproduction has been shown not only for *Phytophthora* [51–53] and *Pythium* [54], but also for Heterokontophyta in general in both oogamy [55] and isogamy [56]. Thus, hybrids formed by sexual cross would be expected to have uniparentally-inherited mitochondria and indeed this has been observed for all *Phytophthora* hybrids characterized to date [13,15,19–21]. Somatic fusion (hybridization) resulting in heterokaryon formation has been forced under laboratory conditions through protoplast fusion between isolates of the same species [57–59]. Interspecific protoplast fusion has not been successful and there is no evidence for this happening naturally [60]. Intraspecific somatic hybridization has been accomplished by the induced fusion of uninucleate zoospores of *P*. *nicotianae* and *P*. *capsici* [61] and *P*. *nicotianae* and *P*. *infestans* [62]. In both cases the presence of DNA of both parents was demonstrated, however mitochondrial DNA was not examined, so it is not known if these hybrids contain mitochondria from both parents. Theoretically, somatic hybridization could be followed by selection or drift amongst the mitochondria resulting in the representation of just one parental type [60,63,64].

In the current study the mitochondrial genes COI and NADH (and by extension the mitochondrial genome) of all hybrid isolates were inherited uniparentally. This pattern of predominantly biparental nuclear inheritance and uniparental mitochondrial inheritance strongly supports sexual hybridization as opposed to somatic hybridization where biparental mitochondrial inheritance could be expected. However, even if, by chance, this group of *Phytophthora* species are capable of somatic hybridization and even if mitochondrial loss does occur leading to a single parental mitochondria, it could have been expected to find two mitochondrial types at least once among the 26 isolates presented here, the 19 presented by [20] or the 112 isolates characterized by [43].

While most of the hybrid isolates characterized here appear to be formed by simple hybridization event between two parental species, several isolates bore the signature of three parents. *Phytophthora* x *alni* also has the genetic material from three parental species and is formed from two sequential hybridization events; the first between unknown parents resulting in *P*. x *multiformis* and the second between *P*. x *multiformis* and *P*. *uniformis* [12]. There is also a report of a natural chimeric yeast containing genetic material from three species, although the authors did not propose a mechanism for how this could have occurred [65]. What cannot be determined from the available data is if the involvement of three parents due to sequential or simultaneous hybridization events. If sequential, the first event would be a biparental hybrid followed by a subsequent event either with another hybrid, or a third parental species that would generate an entity with DNA signatures from three parents (as seen for *P*. x *alni*). If simultaneous, this could have been from a single hybridization event where two antheridia from different species fertilize oogonia of a third species. While, simultaneous hybridization between three *Phytophthora* species has not been recorded it is theoretically possible as multiple antheridia are frequently observed attached to a single oogonium as is the case for clade 6 *Phytophthora* species such as *P*. *thermophila* [35] and *P*. *bilorbang* [66]. Both antheridia and oogonia contain multiple nuclei, near synchronous meiosis occurs in both and the antheridial nucleus migrates into the oogonia through fertilization tubes from one or several antheridia, the male and female gametes aggregate and it is assumed that all other haploid nuclei abort. The oogonium increases in size and the oospore wall is formed. Maturation takes time, but one of the first signs is the fusion of the remaining nuclei [51,67,68].

Of 26 isolates, 15 showed the expected biparental inheritance of alleles at all nuclear loci. However, there were several instances where alleles from both parents were not found (see Fig 6). For example for isolates DH283 and DH284 (A-M hybrids) isolates, both parents were reflected in ITS, only *P*. *amnicola* in HSP, only *P*. *moyootj* in ASF and for BT. Isolate DH283 produced a mixed profile reflecting both parents, while for DH184 only the *P*. *moyootj* allele was recovered. Similarly for isolate DH089 (F-M hybrid), both parents were reflected in ITS, *P*. *fluvialis* in HSP and only *P*. *moyootj* in BT and ASF. These are just a couple of the many examples within the dataset. As yet the ploidy levels of the parent species and hybrid isolates are unknown, so the hybrids may be homoploids, polyploids or aneuploids. The difference in the prevalence of parental alleles could be due to gene or chromosome loss (if they are sterile hybrids) or subsequent sexual reproduction between hybrid isolates or backcrossing with parental isolates resulting in unequal distribution of parental alleles in offspring.

The advantage of the ITS locus in the study of hybrids is its occurrence in the genome as tandem repeat arrays, most likely on several chromosomes and is sensitive to concerted evolution. Over time, recombinant ITS sequences, which bear the signature of both parents, will emerge either through mitotic crossover or meiotic recombination [19,20]. Recombinant ITS sequences were observed for many of the hybrid isolates characterized here (S2 Table). For some isolates such as VHS16115, DH181, DH147 almost all ITS types were recombinant between the two parents while others such as DH106 and DH089 only had the parental alleles. We cannot fully exclude the possibility of PCR mediated recombination [69] however, following the example of [19] in the study of clade 8 *Phytophthora* hybrids, a high fidelity DNA polymerase was used, a technique known to reduce PCR mediated recombination [70]. The extent of recombination could be an expression of the age of the hybrids, with older hybrids having more recombinant ITS sequences [19]. The presence of either both parental species or many recombinant alleles in the current study points to these hybrids being relatively young as the concerted evolution usually leads to the loss of one parental allele or the mixing of the two parental alleles to form of a new distinct ITS type [31,64,71]. Unidirectional concerted evolution is ongoing among *Phytophthora* clade 8 hybrids [19]. Similar processes have been empirically demonstrated for hybrids between the indigenous fungal pathogen *Heterobasidium annosum* and the introduced *H*. *irregulare*. Hybridization has been followed by introgression, admixture and the formation of recombinant alleles with signatures from both parents [72].

### Formation and fertility of clade 6 *Phytophthora* hybrids

All parental species occur in natural waterways and within native vegetation in Western Australia and are considered indigenous; survey data places many of them in same catchments [43]. What is unknown is whether these species were naturally sympatric or naturally allopatric (*i*.*e*. sister taxa found in individual isolated catchments) before human disturbance on a landscape level. If allopatric and they have now been reunited this could account for the apparent ease of interspecies hybridization and, with time, introgression may results in the merging of these species [23,63,73].

As with many clade 6 Phytophthora species [39,74], it is difficult to induce the sexual cycle of the five known parental species under laboratory conditions. Oogonia have been observed for one isolate of *P*. *thermophila* and *P*. *litoralis* is silent A1 [35]. Similarly, one isolate of *P*. *borealis* produced oospores under laboratory conditions [74], and two isolates of the sterile species *P*. *gonapodyides* have produced oogonia when paired [39].

Most clade 6 Phytophthora species are associated with riparian ecosystems and wet soils and although sometimes associated with disease, they have been proposed to play a role in the breakdown of green plant litter [39]. Green litter is still partially resistant to saprophytes and thus a strategy of rapid asexual proliferation would maintain high inoculum levels and help them to outcompete saprophytic organisms. Under this scenario, rapid asexual reproduction is advantageous as asexual zoospores are the infective propagules, while production of long-lived oospores is unnecessary. However, the existence of the hybrids described here, apparently formed through sexual recombination coupled with the rare observations of oogonia under laboratory conditions, suggests that these species are fertile in their natural environment.

From these observations the following scenarios are posed for hybrids characterized here and for the future of these species and hybrids in WA; (1) Parental species proliferate asexually, but capable of sexual reproduction when stimulated by environment conditions or related species. The resultant hybrids are also fertile and capable of backcrossing and may in time lead to the fusion of species or the formation of new species (2) Parental species are capable of sexual reproduction, interspecies hybrids form regularly and easily, the hybrids are sterile, there is no backcrossing. (3) A combination of 1 and 2 whereby hybrid swarms and introgression occur locally (within a catchment, or ephemeral stream), but species integrity as a whole is still maintained across the region.

### Persistence and survival of clade 6 *Phytophthora* hybrids

Most previously reported *Phytophthora* hybrids have arisen in man made environments, such as nurseries and hydroponic systems where the reunion of previously separated but related species has led to the formation of hybrids. In these instances new traits have emerged such as new host specificity or increased pathogenicity [11,19,29]. Most of these hybrids appear to be stable evolutionary lineages and most reproduce sexually, even if somewhat abortive [75]. The naturally formed hybrids characterised here are isolated in high frequency in surveys of streams and waterways and this frequency suggests the parental species are fertile and that there are no barriers to hybridisation. These hybrids appear, for the most part, to be sterile and, given the ephemeral nature of many waterways in WA, perhaps transient hybrid clones. This is certainly highlighted by their poor survival in culture (lack of survival structures). Hybrid sterility would prevent introgression and species fusion and would be an adaptive strategy if the parental species were in fact naturally sympatric.

### Conclusion

Twelve out of a possible 30 two-way hybrid combinations among the six parental species have been characterized. Further sampling in the same environments could result in the discovery of other combinations. I refrain from naming the hybrids for the following reasons; they appear to be continually formed and perhaps transient, some isolates have morphological abnormalities and they do not survive well in culture.

Future experiments should focus on the generation of controlled crosses, but also, and perhaps more importantly, the DNA content of the parental species and the hybrids should be measured as has been done for clade 8 hybrids [19] and more recently for *P*. x *alni* [12]. The development of species specific SNPs or microsatellite markers would also enable the resolution of parental contribution to different hybrid isolates. Such studies would resolve the mechanisms of hybridization among this group of closely related species.

## Supporting Information

S1 Table GenBank Accession numbers of *Phytophthora* isolates considered in this study. (DOCX)

S2 Table Comparison of variable sites in ITS gene region between consensus sequences of parental species and sequences from cloned amplicons of hybrid isolates. (DOCX)
