# Supplementary material for: Molecular Characterization of Natural Hybrids Formed between Five Related Indigenous Clade 6 Phytophthora Species
Source: PLoS One. 2015 Aug 6;10(8):e0134225. doi: 10.1371/journal.pone.0134225 (PMC4527719; doi:10.1371/journal.pone.0134225)
Supplement: S1 Table — (DOCX) [file pone.0134225.s001.docx]

**Table S1.** Genbank Accession numbers of isolates considered in this study

|  |  | Genbank Accession Numbers | | | | | |
| --- | --- | --- | --- | --- | --- | --- | --- |
| **Isolate** | **Species** | **ITS** | **ASF** | **BT** | **HSP** | **COX** | **NADH** |
| CBS131652 | *Phytophthora amnicola* | JQ029956 | JQ936759 | JQ029952 | JQ029944 | JQ029948 | JQ029940 |
| VHS19503 | *P. amnicola* | JQ029958 | JQ936760 | JQ029954 | JQ029946 | JQ029950 | JQ029942 |
| VHS17175 | *P. asparagi* | EU301167 |  | JN547592 | HQ012891 | HQ012844 | JN547680 |
| CBS129424 | *P. fluvialis* | JF701436 | JQ936761 | JN547595 | JF701439 | JF701442 | JN547682 |
| VHS17350 | *P. fluvialis* | EU593261 | JQ936762 | JN547593 | JF701437 | JF701440 | JN547681 |
| CBS309.62 | *P. fragariae* |  | DQ092832 |  |  |  |  |
| MUCC776 | *P. gonapodyides* | JN547620 | JQ936763 | JN547581 | JN547653 | JN547642 | JN547669 |
| P6702 | *P. humicola* | FJ801938 |  | JN935975 | JN935946 | KM883118 | JN936027 |
| VHS16836 | *P. inundata* | HQ012944 |  |  |  | HQ012860 | KM883174 |
| HSA1959 | *P. lacustris* | HQ012956 |  | JN547618 | HQ012924 | HQ012880 | JN547706 |
| VHS17085 | *P. litoralis* | EU593262 | JQ936766 | JN547609 | HQ012909 | HQ012864 | JN547697 |
| CBS127953 | *P. litoralis* | HQ012948 | JQ936765 | JN547611 | HQ012911 | HQ012866 | KF606714 |
| DH180 | *P. amnicola x  P. moyootj* | KM883019 | KM883066 KM883067 | KM883103 | KM883159 | KM883133 | KM883190 |
| DH283 | *P. amnicola x  P. moyootj* | KM883017 | KM883076 KM883077 | KM883109 | KM883165 | KM883139 | KM883196 |
| DH284 | *P. amnicola x  P. moyootj* | KM883018 | KM883078 | KM883110 | KM883166 | KM883140 | KM883197 |
| DH011 | *P. fluvialis x  P. moyootj* | KM883026 | KM883050 KM883051 | KM883093 | KM883149 | KM883124 | KM883180 |
| DH087 | *P. fluvialis x  P. moyootj* | KM883028 | KM883053 KM883054 | KM883095 | KM883151 | KM883126 | KM883182 |
| DH089 | *P. fluvialis x  P. moyootj* | KM883030 | KM883055 | KM883096 | KM883152 | KM883127 | KM883183 |
| DDS3642 | *P. fluvialis x  P. moyootj* | KM883029 | KM883081 KM883082 | KM883112 | KM883168 | KM883123 | KM883199 |
| DH117 | *P. fluvialis* hybrid | KM883031 | KM883059 KM883060 | KM883098 | KM883154 | KM883129 | KM883185 |
| VHS29992 | *P. fluvialis* hybrid | KM883033 | KM883085 KM883086 | KM883117 | KM883173 | KM883144 | KM883204 |
| DH134 | *P. litoralis x  P. moyootj* | KM883035 | KM883061 KM883062 | KM883099 | KM883155 | KM883130 | KM883186 |
| DH147 | *P. litoralis x  P. moyootj* | KM883036 | KM883064 KM883065 | KM883101 | KM883157 | KM883132 | KM883188 |
| VHS16115 | *P. litoralis x  P. moyootj* | KM883037 | KM883087 KM883088 | KM883115 | KM883171 | KM883143 | KM883202 |
| DDS3432 | *P. megasperma* | HQ012949 | JQ936768 | JN547608 |  | HQ012867 | KM883175 |
| VHS16108 | *P. mooyotj* | EU593259 | KM883041 | KJ372302 | KJ396729 | KJ369701 | KJ396680 |
| VHS27218 | *P. mooyotj* | KJ372255 | KM883042 | KJ372303 | KJ396730 | KJ369702 | KJ396681 |
| DH103 | *P. mooyotj* | KJ372255 | KM883040 | KJ372301 | KJ396728 | KJ396700 | KJ396679 |
| DH056 | *P. moyootj* | KM883011 | KM883052 | KM883094 | KM883150 | KM883125 | KM883181 |
| DH137 | *P. moyootj* | KM883013 | KM883063 | KM883100 | KM883156 | KM883131 | KM883187 |
| DH206 | *P. moyootj* | KM883012 | KM883071 | KM883106 | KM883162 | KM883136 | KM883193 |
| BAN-A | *P. moyootj x  P. fluvialis* | KM883032 | KM883043 KM883044 | KM883089 | KM883145 | KM883119 | KM883176 |
| DH181 | *P. moyootj x  P. fluvialis* | KM883024 | KM883068 | KM883104 | KM883160 | KM883134 | KM883191 |
| DH182 | *P. moyootj x  P. fluvialis* | KM883025 | KM883069 KM883070 | KM883105 | KM883161 | KM883135 | KM883192 |
| DH286 | *P. moyootj x  P. fluvialis* | KM883027 | KM883079 KM883080 | KM883111 | KM883167 | KM883141 | KM883198 |
| MUR-C | *P. moyootj x  P. litoralis* | KM883034 | KM883048 KM883049 | KM883092 | KM883148 | KM883122 | KM883179 |
| BAN-B | *P. moyootj x  P. thermophila* | KM883014 | KM883045 KM883046 | KM883090 | KM883146 | KM883120 | KM883177 |
| MUR-A | *P. moyootj x  P. thermophila* | KM883015 | KM883047 | KM883091 | KM883147 | KM883121 | KM883178 |
| VHS2713 | *P. sp.* hybrid | KM883020 | KM883083 KM883084 | KM883113 | KM883169 | KM883142 | KM883200 |
| DH269 | *P. sp. x P.amnicola* | KM883016 | KM883074 KM883075 | KM883108 | KM883164 | KM883138 | KM883195 |
| VHS6595* | *P. taxon PgChlamydo* | EU301159 | JQ936770 | JN547617 | HQ012923 | HQ012879 | JN547704 |
| VHS7474 | *P. thermophila* | HQ012952 | JQ936773 | JN547612 | HQ012915 | HQ012871 | JN547699 |
| VHS13530 | *P. thermophila* | EU301155 | JQ936774 | JN547613 | HQ012916 | HQ012872 | JN547700 |
| DH106 | *P. thermophila* hybrid | KM883039 | KM883056 KM883057 KM883058 | KM883097 | KM883153 | KM883128 | KM883184 |
| DH150 | *P. thermophila x P. amnicola* | KM883021 | JQ936779 JQ936792 | KM883102 | KM883158 | JQ936803 | KM883189 |
| VHS5185 | *P. thermophila x P. amnicola* | KM883023 | JQ936782 JQ936795 | KM883114 | KM883170 | JQ936806 | KM883201 |
| VHS22715 | *P. thermophila x P. amnicola* | KM883022 | JQ936783 JQ936796 | KM883116 | KM883172 | JQ936807 | KM883203 |
| DH265 | *P. thermophila x P.moyootj* | KM883038 | KM883072 KM883073 | KM883107 | KM883163 | KM883137 | KM883194 |
