# Supplementary material for: Molecular Characterization of Natural Hybrids Formed between Five Related Indigenous Clade 6 Phytophthora Species
Source: PLoS One. 2015 Aug 6;10(8):e0134225. doi: 10.1371/journal.pone.0134225 (PMC4527719; doi:10.1371/journal.pone.0134225)
Supplement: S2 Table — (DOCX) [file pone.0134225.s002.docx]

**Table S2.** Comparison of variable sites in ITS gene region between consensus sequences of parental species and sequences from cloned amplicons of hybrid isolates

| Species/ Isolate | N^1^ | ITS polymorphisms | | | | | | | | | | | | | | | | | | | | | | | | | | | | | | | | | | | |
| --- | --- | --- | --- | --- | --- | --- | --- | --- | --- | --- | --- | --- | --- | --- | --- | --- | --- | --- | --- | --- | --- | --- | --- | --- | --- | --- | --- | --- | --- | --- | --- | --- | --- | --- | --- | --- | --- |
|  |  | **41** | **44** | **45** | **47** | **59** | **61** | **71** | **111** | **162** | **171** | **173** | **176** | **177** | **178** | **180** | **182** | **184** | **476** | **477** | **518** | **521** | **555** | **583** | **654** | **676** | **678** | **703** | **721** | **746** | **750** | **751** | **756** | **756** | **788** | **791** | **814** |
| ***P. amnicola*** |  | **C** | **T** | **A** | **T** | **T** | **C** | **C** | **G** | **C** | **T** | **C** | **T** | **C** | **T** | **A** | **A** | **T** | **G** | **T** | **T** | **T** | **C** | **A** | **G** | **G** | **A** | **G** | **C** | **A** | **T** | **-** | **C** | **G** | **C** | **T** | **T** |
| ***P. amnicola*** |  | **C** | **T** | **A** | **T** | **T** | **C** | **C** | **G** | **C** | **T** | **C** | **T** | **C** | **T** | **A** | **A** | **T** | **G** | **T** | **T** | **T** | **C** | **A** | **G** | **G** | **A** | **G** | **C** | **A** | **C** | **T** | **T** | **T** | **C** | **T** | **T** |
| ***P. fluvialis*** |  | **T** | **T** | **T** | **A** | **C** | **C** | **T** | **G** | **-** | **T** | **A** | **C** | **A** | **A** | **T** | **T** | **T** | **C** | **G** | **T** | **T** | **T** | **T** | **T** | **A** | **G** | **R** | **G** | **T** | **T** | **-** | **C** | **G** | **T** | **T** | **C** |
| ***P. litoralis*** |  | **T** | **-** | **T** | **A** | **T** | **T** | **T** | **A** | **C** | **-** | **C** | **T** | **C** | **T** | **-** | **A** | **T** | **C** | **G** | **C** | **C** | **T** | **A** | **G** | **G** | **A** | **G** | **C** | **A** | **C** | **-** | **C** | **G** | **C** | **T** | **T** |
| ***P. mooyotj*** |  | **T** | **T** | **T** | **A** | **T** | **C** | **T** | **G** | **C** | **-** | **C** | **T** | **C** | **T** | **A** | **A** | **T** | **C** | **G** | **T** | **T** | **T** | **A** | **G** | **A** | **G** | **A** | **C** | **A** | **T** | **-** | **C** | **G** | **C** | **T** | **T** |
| ***P. thermophila*** |  | **T** | **A** | **A** | **T** | **C** | **C** | **T** | **G** | **C** | **-** | **C** | **T** | **C** | **T** | **A** | **A** | **A** | **C** | **G** | **T** | **T** | **T** | **A** | **G** | **G** | **A** | **G** | **C** | **G** | **C** | **-** | **C** | **G** | **C** | **T** | **T** |
|  |  |  |  |  |  |  |  |  |  |  |  |  |  |  |  |  |  |  |  |  |  |  |  |  |  |  |  |  |  |  |  |  |  |  |  |  |  |
| **DH269** | **3** | **C** | **T** | **A** | **T** | **T** | **C** | **C** | **G** | **C** | **T** | **C** | **T** | **C** | **T** | **A** | **A** | **T** | **G** | **T** | **T** | **T** | **C** | **A** | **G** | **G** | **G** | **G** | **C** | **A** | **C** | **T** | **T** | **T** | **G** | **T** | **T** |
|  | **1** | **C** | **T** | **A** | **T** | **T** | **C** | **C** | **G** | **C** | **-** | **C** | **T** | **C** | **T** | **A** | **C** | **T** | **C** | **G** | **T** | **T** | **T** | **A** | **G** | **A** | **G** | **G** | **C** | **A** | **T** | **-** | **C** | **G** | **C** | **T** | **T** |
|  | **1** | **C** | **T** | **A** | **T** | **T** | **C** | **C** | **G** | **C** | **T** | **C** | **T** | **C** | **T** | **A** | **C** | **T** | **C** | **G** | **T** | **T** | **T** | **A** | **G** | **A** | **G** | **A** | **C** | **A** | **T** | **-** | **C** | **G** | **C** | **T** | **T** |
|  | **4** | **T** | **T** | **T** | **A** | **C** | **C** | **T** | **G** | **-** | **-** | **C** | **T** | **C** | **T** | **A** | **C** | **T** | **C** | **G** | **T** | **T** | **T** | **A** | **G** | **A** | **G** | **A** | **C** | **A** | **T** | **-** | **C** | **G** | **C** | **T** | **T** |
|  |  |  |  |  |  |  |  |  |  |  |  |  |  |  |  |  |  |  |  |  |  |  |  |  |  |  |  |  |  |  |  |  |  |  |  |  |  |
| **DH283** | **2** | **C** | **T** | **A** | **T** | **T** | **C** | **C** | **G** | **C** | **T** | **C** | **T** | **C** | **T** | **A** | **A** | **T** | **G** | **T** | **T** | **T** | **C** | **A** | **G** | **G** | **A** | **G** | **C** | **A** | **T** | **-** | **C** | **G** | **C** | **T** | **T** |
|  | **1** | **C** | **T** | **A** | **T** | **T** | **C** | **C** | **G** | **C** | **T** | **C** | **T** | **C** | **T** | **A** | **A** | **T** | **G** | **T** | **T** | **T** | **C** | **A** | **G** | **G** | **A** | **G** | **C** | **A** | **C** | **T** | **T** | **T** | **C** | **T** | **T** |
|  | **1** | **C** | **T** | **A** | **T** | **T** | **C** | **C** | **G** | **C** | **-** | **C** | **T** | **C** | **T** | **A** | **A** | **T** | **C** | **G** | **T** | **T** | **T** | **A** | **G** | **A** | **G** | **A** | **C** | **A** | **T** | **-** | **C** | **G** | **C** | **T** | **T** |
|  | **1** | **T** | **T** | **T** | **A** | **T** | **T** | **C** | **G** | **C** | **C** | **C** | **T** | **C** | **T** | **A** | **A** | **T** | **C** | **G** | **T** | **T** | **T** | **A** | **G** | **A** | **G** | **A** | **C** | **A** | **T** | **-** | **C** | **G** | **C** | **T** | **T** |
|  | **4** | **T** | **T** | **T** | **A** | **T** | **T** | **C** | **G** | **C** | **-** | **C** | **T** | **C** | **T** | **A** | **A** | **T** | **C** | **G** | **T** | **T** | **T** | **A** | **G** | **A** | **A** | **A** | **C** | **A** | **T** | **-** | **C** | **G** | **C** | **T** | **T** |
|  |  |  |  |  |  |  |  |  |  |  |  |  |  |  |  |  |  |  |  |  |  |  |  |  |  |  |  |  |  |  |  |  |  |  |  |  |  |
| **DH284** | **1** | **C** | **T** | **A** | **T** | **T** | **C** | **C** | **G** | **C** | **T** | **C** | **T** | **C** | **T** | **A** | **A** | **T** | **G** | **T** | **T** | **T** | **C** | **A** | **G** | **G** | **A** | **G** | **C** | **A** | **T** | **-** | **C** | **G** | **C** | **T** | **T** |
|  | **1** | **C** | **T** | **A** | **T** | **T** | **C** | **C** | **G** | **C** | **-** | **C** | **T** | **C** | **T** | **A** | **A** | **T** | **G** | **T** | **T** | **T** | **C** | **A** | **G** | **G** | **A** | **A** | **C** | **A** | **T** | **-** | **C** | **G** | **C** | **T** | **T** |
|  | **1** | **T** | **T** | **A** | **A** | **T** | **C** | **C** | **G** | **C** | **-** | **C** | **T** | **C** | **T** | **A** | **A** | **T** | **G** | **T** | **T** | **T** | **C** | **A** | **G** | **G** | **A** | **G** | **C** | **A** | **T** | **-** | **C** | **G** | **T** | **T** | **T** |
|  | **1** | **T** | **T** | **A** | **A** | **T** | **C** | **C** | **G** | **C** | **T** | **C** | **T** | **C** | **T** | **A** | **A** | **T** | **G** | **T** | **T** | **T** | **C** | **A** | **G** | **G** | **A** | **G** | **C** | **A** | **T** | **-** | **C** | **G** | **C** | **T** | **T** |
|  | **2** | **T** | **T** | **A** | **A** | **T** | **C** | **C** | **G** | **C** | **-** | **C** | **T** | **C** | **T** | **A** | **A** | **T** | **C** | **G** | **T** | **T** | **T** | **A** | **G** | **A** | **A** | **A** | **C** | **A** | **T** | **-** | **C** | **G** | **C** | **T** | **T** |
|  | **1** | **T** | **T** | **A** | **A** | **T** | **C** | **C** | **G** | **C** | **-** | **C** | **T** | **C** | **T** | **A** | **A** | **T** | **C** | **G** | **T** | **T** | **T** | **A** | **G** | **A** | **A** | **A** | **C** | **A** | **T** | **T** | **T** | **T** | **C** | **T** | **T** |
|  |  |  |  |  |  |  |  |  |  |  |  |  |  |  |  |  |  |  |  |  |  |  |  |  |  |  |  |  |  |  |  |  |  |  |  |  |  |
| **DH180** | **1** | **C** | **T** | **A** | **T** | **T** | **C** | **C** | **G** | **C** | **T** | **C** | **T** | **C** | **T** | **A** | **A** | **T** | **G** | **T** | **T** | **G** | **C** | **A** | **G** | **G** | **A** | **G** | **C** | **A** | **C** | **T** | **T** | **T** | **C** | **T** | **T** |
|  | **2** | **T** | **T** | **T** | **A** | **T** | **C** | **C** | **G** | **C** | **-** | **C** | **T** | **C** | **T** | **A** | **A** | **T** | **C** | **G** | **T** | **G** | **T** | **A** | **G** | **A** | **A** | **A** | **C** | **A** | **T** | **-** | **C** | **G** | **C** | **T** | **T** |
|  | **1** | **C** | **T** | **A** | **T** | **T** | **C** | **C** | **G** | **C** | **T** | **C** | **T** | **C** | **T** | **A** | **A** | **T** | **C** | **G** | **T** | **G** | **T** | **A** | **G** | **G** | **A** | **G** | **C** | **A** | **T** | **-** | **C** | **G** | **C** | **T** | **T** |
|  | **2** | **C** | **T** | **A** | **T** | **T** | **C** | **C** | **G** | **C** | **T** | **C** | **T** | **C** | **T** | **A** | **A** | **T** | **C** | **G** | **T** | **G** | **T** | **A** | **G** | **A** | **A** | **A** | **C** | **A** | **T** | **-** | **C** | **G** | **C** | **T** | **T** |
|  | **15** | **T** | **T** | **T** | **A** | **T** | **C** | **T** | **G** | **C** | **-** | **C** | **T** | **C** | **T** | **A** | **A** | **T** | **C** | **G** | **T** | **G** | **T** | **A** | **G** | **A** | **A** | **A** | **C** | **A** | **T** | **-** | **C** | **G** | **C** | **T** | **T** |
|  |  |  |  |  |  |  |  |  |  |  |  |  |  |  |  |  |  |  |  |  |  |  |  |  |  |  |  |  |  |  |  |  |  |  |  |  |  |
| **VHS2713** | **17** | **C** | **T** | **A** | **T** | **T** | **C** | **C** | **G** | **C** | **T** | **C** | **T** | **C** | **T** | **A** | **A** | **T** | **G** | **T** | **T** | **T** | **C** | **A** | **G** | **G** | **A** | **G** | **C** | **A** | **T** | **-** | **C** | **G** | **G** | **T** | **T** |
|  | **5** | **C** | **T** | **A** | **T** | **T** | **C** | **C** | **G** | **C** | **T** | **C** | **T** | **C** | **T** | **A** | **A** | **T** | **G** | **T** | **T** | **T** | **T** | **A** | **G** | **A** | **G** | **A** | **C** | **A** | **T** | **-** | **C** | **G** | **C** | **T** | **C** |
|  | **6** | **C** | **T** | **A** | **T** | **T** | **C** | **C** | **G** | **C** | **T** | **C** | **T** | **C** | **T** | **A** | **A** | **T** | **C** | **G** | **T** | **T** | **T** | **A** | **G** | **A** | **G** | **A** | **C** | **A** | **T** | **-** | **C** | **G** | **C** | **T** | **C** |
|  | **1** | **T** | **T** | **T** | **A** | **C** | **C** | **T** | **G** | **C** | **T** | **C** | **T** | **C** | **T** | **A** | **A** | **T** | **G** | **T** | **T** | **T** | **C** | **A** | **G** | **G** | **A** | **G** | **C** | **A** | **T** | **-** | **C** | **G** | **G** | **T** | **T** |
|  | **1** | **T** | **T** | **T** | **A** | **C** | **C** | **T** | **G** | **C** | **T** | **C** | **T** | **C** | **T** | **A** | **A** | **T** | **C** | **G** | **T** | **T** | **T** | **A** | **G** | **A** | **G** | **A** | **C** | **A** | **T** | **-** | **C** | **G** | **C** | **T** | **T** |
|  | **2** | **C** | **T** | **A** | **T** | **T** | **C** | **C** | **G** | **-** | **-** | **C** | **T** | **C** | **T** | **A** | **C** | **T** | **C** | **G** | **T** | **T** | **T** | **A** | **G** | **A** | **G** | **A** | **C** | **A** | **T** | **-** | **C** | **G** | **G** | **T** | **T** |
|  | **2** | **T** | **T** | **T** | **A** | **C** | **C** | **T** | **G** | **-** | **-** | **C** | **T** | **C** | **T** | **A** | **C** | **T** | **G** | **T** | **T** | **T** | **C** | **A** | **G** | **A** | **G** | **A** | **C** | **A** | **T** | **-** | **C** | **G** | **C** | **T** | **C** |
|  | **6** | **T** | **T** | **T** | **A** | **C** | **C** | **T** | **G** | **-** | **-** | **C** | **T** | **C** | **T** | **A** | **C** | **T** | **C** | **G** | **T** | **T** | **T** | **A** | **G** | **G** | **A** | **G** | **C** | **A** | **T** | **-** | **C** | **G** | **C** | **T** | **C** |
|  | **5** | **T** | **T** | **T** | **A** | **C** | **C** | **T** | **G** | **-** | **-** | **C** | **T** | **C** | **T** | **A** | **C** | **T** | **G** | **T** | **T** | **T** | **C** | **A** | **G** | **G** | **A** | **G** | **C** | **A** | **T** | **-** | **C** | **G** | **G** | **T** | **T** |
|  | **1** | **T** | **T** | **T** | **A** | **C** | **C** | **T** | **G** | **-** | **-** | **C** | **T** | **C** | **T** | **A** | **C** | **T** | **C** | **G** | **T** | **T** | **C** | **A** | **G** | **G** | **A** | **G** | **C** | **A** | **T** | **-** | **C** | **G** | **G** | **T** | **T** |
|  | **34** | **T** | **T** | **T** | **A** | **C** | **C** | **T** | **G** | **-** | **-** | **C** | **T** | **C** | **T** | **A** | **C** | **T** | **C** | **G** | **T** | **T** | **T** | **A** | **G** | **A** | **G** | **A** | **C** | **A** | **T** | **-** | **C** | **G** | **C** | **T** | **C** |
|  |  |  |  |  |  |  |  |  |  |  |  |  |  |  |  |  |  |  |  |  |  |  |  |  |  |  |  |  |  |  |  |  |  |  |  |  |  |
| **DH150** | **8** | **C** | **T** | **A** | **T** | **T** | **C** | **C** | **G** | **C** | **T** | **C** | **T** | **C** | **T** | **A** | **A** | **T** | **G** | **T** | **T** | **T** | **C** | **A** | **G** | **G** | **A** | **G** | **C** | **A** | **T** | **-** | **C** | **G** | **C** | **T** | **T** |
|  | **1** | **T** | **A** | **A** | **T** | **C** | **C** | **T** | **G** | **C** | **-** | **C** | **T** | **C** | **T** | **A** | **A** | **A** | **C** | **G** | **T** | **T** | **C** | **A** | **G** | **G** | **A** | **G** | **C** | **A** | **T** | **-** | **C** | **G** | **C** | **T** | **T** |
|  |  |  |  |  |  |  |  |  |  |  |  |  |  |  |  |  |  |  |  |  |  |  |  |  |  |  |  |  |  |  |  |  |  |  |  |  |  |
| **VHS22715** | **1** | **C** | **T** | **A** | **T** | **T** | **C** | **C** | **G** | **C** | **T** | **C** | **T** | **C** | **T** | **A** | **A** | **T** | **C** | **G** | **T** | **T** | **T** | **A** | **G** | **G** | **A** | **G** | **C** | **A** | **T** | **-** | **C** | **G** | **C** | **T** | **T** |
|  | **2** | **C** | **T** | **A** | **T** | **T** | **C** | **C** | **G** | **C** | **T** | **C** | **T** | **C** | **T** | **A** | **A** | **T** | **C** | **G** | **T** | **T** | **T** | **A** | **G** | **G** | **A** | **G** | **C** | **G** | **C** | **-** | **C** | **G** | **C** | **T** | **T** |
|  | **7** | **C** | **T** | **A** | **T** | **T** | **C** | **C** | **G** | **C** | **T** | **C** | **T** | **C** | **T** | **A** | **A** | **T** | **G** | **T** | **T** | **T** | **C** | **A** | **G** | **G** | **A** | **G** | **C** | **A** | **T** | **-** | **C** | **G** | **C** | **T** | **T** |
|  |  |  |  |  |  |  |  |  |  |  |  |  |  |  |  |  |  |  |  |  |  |  |  |  |  |  |  |  |  |  |  |  |  |  |  |  |  |
| **VHS5185** | **1** | **C** | **T** | **A** | **T** | **T** | **C** | **C** | **G** | **C** | **T** | **C** | **T** | **C** | **T** | **A** | **A** | **T** | **C** | **G** | **T** | **T** | **T** | **A** | **G** | **G** | **A** | **G** | **C** | **G** | **C** | **-** | **C** | **G** | **C** | **T** | **T** |
|  | **1** | **C** | **T** | **A** | **T** | **T** | **C** | **C** | **G** | **C** | **T** | **C** | **T** | **C** | **T** | **A** | **A** | **T** | **C** | **G** | **T** | **T** | **T** | **A** | **G** | **G** | **A** | **G** | **C** | **A** | **T** | **-** | **C** | **G** | **T** | **T** | **T** |
|  | **1** | **C** | **T** | **A** | **T** | **T** | **C** | **C** | **G** | **C** | **T** | **C** | **T** | **C** | **T** | **A** | **A** | **T** | **C** | **G** | **T** | **T** | **C** | **A** | **G** | **G** | **A** | **G** | **C** | **A** | **T** | **-** | **C** | **G** | **T** | **T** | **T** |
|  | **1** | **C** | **T** | **A** | **T** | **C** | **C** | **T** | **G** | **C** | **-** | **C** | **T** | **C** | **T** | **A** | **A** | **A** | **C** | **G** | **T** | **T** | **T** | **A** | **G** | **G** | **A** | **G** | **C** | **A** | **T** | **-** | **C** | **G** | **T** | **T** | **T** |
|  | **20** | **C** | **T** | **A** | **T** | **T** | **C** | **C** | **G** | **C** | **T** | **C** | **T** | **C** | **T** | **A** | **A** | **T** | **G** | **T** | **T** | **T** | **C** | **A** | **G** | **G** | **A** | **G** | **C** | **A** | **T** | **-** | **C** | **G** | **T** | **T** | **T** |
|  |  |  |  |  |  |  |  |  |  |  |  |  |  |  |  |  |  |  |  |  |  |  |  |  |  |  |  |  |  |  |  |  |  |  |  |  |  |
| **DH181** | **2** | **T** | **T** | **T** | **A** | **C** | **C** | **T** | **G** | **-** | **T** | **A** | **C** | **A** | **A** | **T** | **T** | **T** | **C** | **G** | **T** | **T** | **T** | **T** | **T** | **A** | **G** | **G** | **G** | **T** | **T** | **-** | **C** | **G** | **T** | **T** | **C** |
|  | **3** | **T** | **T** | **T** | **A** | **T** | **C** | **T** | **G** | **C** | **-** | **C** | **T** | **C** | **T** | **A** | **A** | **T** | **C** | **G** | **T** | **T** | **T** | **A** | **G** | **A** | **G** | **A** | **G** | **A** | **T** | **-** | **C** | **G** | **C** | **T** | **T** |
|  | **2** | **T** | **T** | **T** | **A** | **T** | **C** | **T** | **G** | **C** | **-** | **C** | **T** | **C** | **T** | **A** | **A** | **T** | **C** | **G** | **T** | **T** | **T** | **A** | **T** | **A** | **G** | **G** | **G** | **T** | **T** | **-** | **C** | **G** | **T** | **A** | **C** |
|  | **1** | **T** | **T** | **T** | **A** | **C** | **C** | **T** | **G** | **C** | **-** | **C** | **T** | **C** | **T** | **A** | **A** | **T** | **C** | **G** | **T** | **T** | **T** | **A** | **G** | **A** | **G** | **A** | **G** | **A** | **T** | **-** | **C** | **G** | **C** | **T** | **T** |
|  | **1** | **T** | **T** | **T** | **A** | **C** | **C** | **T** | **G** | **-** | **T** | **A** | **C** | **A** | **A** | **A** | **A** | **T** | **C** | **G** | **T** | **T** | **T** | **A** | **G** | **A** | **G** | **A** | **G** | **A** | **T** | **-** | **C** | **G** | **C** | **T** | **T** |
|  | **1** | **T** | **T** | **T** | **A** | **C** | **C** | **T** | **G** | **-** | **T** | **A** | **C** | **A** | **A** | **A** | **T** | **T** | **C** | **G** | **T** | **T** | **T** | **A** | **G** | **A** | **G** | **A** | **G** | **A** | **T** | **-** | **C** | **G** | **C** | **T** | **T** |
|  |  |  |  |  |  |  |  |  |  |  |  |  |  |  |  |  |  |  |  |  |  |  |  |  |  |  |  |  |  |  |  |  |  |  |  |  |  |
| **DH182** | **4** | **T** | **T** | **T** | **A** | **C** | **C** | **T** | **G** | **-** | **T** | **A** | **C** | **A** | **A** | **A** | **T** | **T** | **C** | **G** | **T** | **T** | **T** | **T** | **T** | **A** | **G** | **G** | **G** | **T** | **T** | **-** | **C** | **G** | **T** | **A** | **C** |
|  | **2** | **T** | **T** | **T** | **A** | **T** | **C** | **T** | **G** | **C** | **-** | **C** | **T** | **C** | **T** | **A** | **A** | **T** | **C** | **G** | **T** | **T** | **T** | **A** | **G** | **A** | **G** | **A** | **C** | **A** | **T** | **-** | **C** | **G** | **C** | **T** | **T** |
|  | **3** | **T** | **T** | **T** | **A** | **C** | **C** | **T** | **G** | **-** | **T** | **A** | **C** | **A** | **A** | **A** | **T** | **T** | **C** | **G** | **T** | **T** | **T** | **T** | **G** | **A** | **G** | **A** | **C** | **A** | **T** | **-** | **C** | **G** | **C** | **T** | **T** |
|  | **1** | **T** | **T** | **T** | **A** | **T** | **C** | **T** | **G** | **-** | **T** | **A** | **C** | **A** | **A** | **A** | **T** | **T** | **C** | **G** | **T** | **T** | **T** | **A** | **G** | **A** | **G** | **A** | **C** | **A** | **T** | **-** | **C** | **G** | **C** | **T** | **T** |
|  |  |  |  |  |  |  |  |  |  |  |  |  |  |  |  |  |  |  |  |  |  |  |  |  |  |  |  |  |  |  |  |  |  |  |  |  |  |
| **DH011** | **5** | **T** | **T** | **T** | **A** | **C** | **C** | **T** | **G** | **-** | **T** | **A** | **C** | **A** | **A** | **A** | **T** | **T** | **C** | **G** | **T** | **T** | **T** | **T** | **T** | **A** | **G** | **A** | **G** | **T** | **T** | **-** | **C** | **G** | **T** | **T** | **C** |
|  | **4** | **T** | **T** | **T** | **A** | **T** | **C** | **T** | **G** | **C** | **-** | **C** | **T** | **C** | **T** | **A** | **A** | **T** | **C** | **G** | **T** | **T** | **T** | **A** | **G** | **A** | **G** | **A** | **C** | **A** | **T** | **-** | **C** | **G** | **C** | **T** | **T** |
|  | **1** | **T** | **T** | **T** | **A** | **T** | **C** | **T** | **G** | **C** | **-** | **C** | **T** | **C** | **T** | **A** | **A** | **T** | **C** | **G** | **T** | **T** | **T** | **A** | **T** | **A** | **G** | **A** | **G** | **T** | **T** | **-** | **C** | **G** | **T** | **T** | **C** |
|  |  |  |  |  |  |  |  |  |  |  |  |  |  |  |  |  |  |  |  |  |  |  |  |  |  |  |  |  |  |  |  |  |  |  |  |  |  |
| **DH286** | **2** | **T** | **T** | **T** | **A** | **T** | **C** | **T** | **G** | **-** | **T** | **A** | **C** | **A** | **A** | **T** | **T** | **T** | **C** | **G** | **T** | **T** | **T** | **T** | **T** | **A** | **G** | **A** | **G** | **T** | **T** | **-** | **C** | **G** | **T** | **A** | **C** |
|  | **6** | **T** | **T** | **C** | **A** | **T** | **C** | **T** | **G** | **C** | **-** | **C** | **T** | **C** | **T** | **A** | **A** | **T** | **C** | **G** | **T** | **T** | **T** | **A** | **G** | **A** | **G** | **A** | **C** | **A** | **T** | **-** | **C** | **G** | **C** | **T** | **T** |
|  | **1** | **T** | **T** | **T** | **A** | **T** | **C** | **T** | **G** | **C** | **-** | **C** | **T** | **C** | **T** | **A** | **A** | **T** | **C** | **G** | **T** | **T** | **T** | **A** | **G** | **A** | **G** | **A** | **C** | **T** | **T** | **-** | **C** | **G** | **C** | **T** | **T** |
|  | **1** | **T** | **T** | **T** | **A** | **T** | **C** | **T** | **G** | **C** | **-** | **C** | **T** | **C** | **T** | **A** | **A** | **T** | **C** | **G** | **T** | **T** | **T** | **A** | **T** | **A** | **G** | **G** | **G** | **T** | **T** | **-** | **C** | **G** | **C** | **T** | **T** |
|  |  |  |  |  |  |  |  |  |  |  |  |  |  |  |  |  |  |  |  |  |  |  |  |  |  |  |  |  |  |  |  |  |  |  |  |  |  |
| **DH087** | **1** | **T** | **T** | **T** | **A** | **C** | **C** | **T** | **G** | **-** | **T** | **A** | **C** | **A** | **A** | **A** | **T** | **T** | **C** | **G** | **T** | **T** | **T** | **T** | **T** | **A** | **G** | **A** | **G** | **T** | **T** | **-** | **C** | **G** | **T** | **T** | **C** |
|  | **7** | **T** | **T** | **T** | **A** | **T** | **C** | **T** | **G** | **C** | **-** | **C** | **T** | **C** | **T** | **A** | **A** | **T** | **C** | **G** | **T** | **T** | **T** | **A** | **G** | **A** | **G** | **A** | **C** | **A** | **C** | **-** | **C** | **G** | **C** | **T** | **T** |
|  | **1** | **T** | **T** | **T** | **A** | **C** | **C** | **T** | **G** | **-** | **T** | **A** | **C** | **A** | **A** | **A** | **T** | **T** | **C** | **G** | **T** | **T** | **T** | **A** | **G** | **A** | **G** | **A** | **C** | **A** | **T** | **-** | **C** | **G** | **G** | **T** | **T** |
|  | **1** | **T** | **T** | **T** | **A** | **C** | **C** | **T** | **G** | **-** | **T** | **A** | **C** | **A** | **A** | **T** | **T** | **T** | **C** | **G** | **T** | **T** | **T** | **T** | **T** | **A** | **G** | **A** | **C** | **A** | **T** | **-** | **C** | **G** | **G** | **T** | **T** |
|  |  |  |  |  |  |  |  |  |  |  |  |  |  |  |  |  |  |  |  |  |  |  |  |  |  |  |  |  |  |  |  |  |  |  |  |  |  |
| **DH117** | **3** | **T** | **T** | **T** | **A** | **C** | **C** | **T** | **G** | **-** | **T** | **A** | **C** | **A** | **A** | **T** | **T** | **T** | **C** | **G** | **T** | **T** | **T** | **T** | **T** | **A** | **G** | **G** | **G** | **T** | **T** | **-** | **C** | **G** | **T** | **A** | **C** |
|  | **5** | **T** | **T** | **T** | **A** | **T** | **C** | **T** | **G** | **C** | **-** | **C** | **T** | **C** | **T** | **A** | **A** | **T** | **C** | **G** | **T** | **T** | **T** | **A** | **G** | **A** | **G** | **A** | **C** | **A** | **T** | **-** | **C** | **G** | **C** | **T** | **T** |
|  | **1** | **T** | **T** | **T** | **A** | **C** | **C** | **T** | **G** | **-** | **T** | **A** | **C** | **A** | **A** | **A** | **T** | **T** | **C** | **G** | **T** | **T** | **T** | **T** | **T** | **A** | **G** | **A** | **G** | **T** | **T** | **-** | **C** | **G** | **C** | **T** | **T** |
|  | **1** | **T** | **T** | **T** | **A** | **T** | **C** | **T** | **G** | **C** | **-** | **C** | **T** | **C** | **T** | **A** | **A** | **T** | **C** | **G** | **T** | **T** | **T** | **A** | **G** | **A** | **G** | **A** | **C** | **A** | **T** | **-** | **C** | **G** | **T** | **T** | **C** |
|  |  |  |  |  |  |  |  |  |  |  |  |  |  |  |  |  |  |  |  |  |  |  |  |  |  |  |  |  |  |  |  |  |  |  |  |  |  |
| **DDS3641** | **3** | **T** | **T** | **T** | **T** | **C** | **C** | **T** | **G** | **-** | **T** | **A** | **C** | **A** | **A** | **T** | **T** | **T** | **C** | **G** | **T** | **T** | **T** | **T** | **T** | **A** | **G** | **G** | **G** | **T** | **T** | **-** | **C** | **G** | **T** | **A** | **C** |
|  | **2** | **T** | **T** | **T** | **T** | **C** | **C** | **T** | **G** | **-** | **T** | **A** | **C** | **A** | **A** | **A** | **T** | **T** | **C** | **G** | **T** | **T** | **T** | **T** | **T** | **A** | **G** | **A** | **C** | **A** | **T** | **-** | **C** | **G** | **C** | **T** | **T** |
|  | **2** | **T** | **T** | **T** | **T** | **T** | **C** | **T** | **G** | **C** | **-** | **C** | **T** | **C** | **T** | **A** | **A** | **T** | **C** | **G** | **T** | **T** | **T** | **T** | **T** | **A** | **G** | **A** | **C** | **A** | **T** | **-** | **C** | **G** | **C** | **T** | **T** |
|  | **2** | **T** | **T** | **T** | **T** | **C** | **C** | **T** | **G** | **C** | **T** | **A** | **C** | **A** | **A** | **T** | **T** | **T** | **C** | **G** | **T** | **T** | **T** | **T** | **T** | **A** | **G** | **A** | **G** | **T** | **T** | **-** | **C** | **G** | **T** | **A** | **C** |
|  |  |  |  |  |  |  |  |  |  |  |  |  |  |  |  |  |  |  |  |  |  |  |  |  |  |  |  |  |  |  |  |  |  |  |  |  |  |
| **DH089** | **2** | **T** | **T** | **T** | **A** | **T** | **C** | **T** | **G** | **C** | **-** | **C** | **T** | **C** | **T** | **A** | **A** | **T** | **C** | **G** | **T** | **T** | **T** | **A** | **G** | **A** | **G** | **A** | **C** | **A** | **T** | **-** | **C** | **G** | **C** | **T** | **T** |
|  | **18** | **T** | **T** | **T** | **A** | **C** | **C** | **T** | **G** | **-** | **T** | **A** | **C** | **A** | **A** | **A** | **T** | **T** | **C** | **G** | **T** | **T** | **T** | **T** | **T** | **A** | **G** | **A** | **G** | **T** | **T** | **-** | **C** | **G** | **T** | **A** | **C** |
|  |  |  |  |  |  |  |  |  |  |  |  |  |  |  |  |  |  |  |  |  |  |  |  |  |  |  |  |  |  |  |  |  |  |  |  |  |  |
| **DH265** | **4** | **T** | **T** | **T** | **A** | **T** | **C** | **T** | **G** | **C** | **-** | **C** | **T** | **C** | **T** | **A** | **A** | **T** | **C** | **G** | **T** | **T** | **T** | **A** | **G** | **A** | **G** | **A** | **C** | **A** | **T** | **-** | **C** | **G** | **C** | **T** | **T** |
|  | **3** | **T** | **A** | **A** | **T** | **C** | **C** | **T** | **G** | **C** | **-** | **C** | **T** | **C** | **T** | **A** | **A** | **A** | **C** | **G** | **T** | **T** | **T** | **A** | **G** | **G** | **A** | **G** | **C** | **G** | **C** | **-** | **C** | **G** | **C** | **T** | **T** |
|  | **1** | **T** | **A** | **A** | **T** | **C** | **C** | **T** | **G** | **C** | **-** | **C** | **T** | **C** | **T** | **A** | **A** | **A** | **C** | **G** | **T** | **T** | **T** | **A** | **G** | **A** | **G** | **A** | **C** | **A** | **T** | **-** | **C** | **G** | **C** | **T** | **T** |
|  | **1** | **T** | **T** | **T** | **A** | **T** | **C** | **T** | **G** | **C** | **-** | **C** | **T** | **C** | **T** | **A** | **A** | **T** | **C** | **G** | **T** | **T** | **T** | **A** | **G** | **G** | **A** | **A** | **C** | **G** | **C** | **-** | **C** | **G** | **C** | **T** | **T** |
|  |  |  |  |  |  |  |  |  |  |  |  |  |  |  |  |  |  |  |  |  |  |  |  |  |  |  |  |  |  |  |  |  |  |  |  |  |  |
| **DH106** | **9** | **T** | **T** | **T** | **A** | **T** | **C** | **T** | **G** | **C** | **-** | **C** | **T** | **C** | **T** | **A** | **A** | **T** | **C** | **G** | **T** | **T** | **T** | **A** | **G** | **A** | **G** | **A** | **C** | **A** | **T** | **-** | **C** | **G** | **C** | **T** | **T** |
|  | **1** | **T** | **A** | **A** | **T** | **C** | **C** | **T** | **G** | **C** | **-** | **C** | **T** | **C** | **T** | **A** | **A** | **A** | **C** | **G** | **T** | **T** | **T** | **A** | **G** | **G** | **A** | **G** | **C** | **G** | **C** | **-** | **C** | **G** | **C** | **T** | **T** |
|  |  |  |  |  |  |  |  |  |  |  |  |  |  |  |  |  |  |  |  |  |  |  |  |  |  |  |  |  |  |  |  |  |  |  |  |  |  |
| **DH134** | **1** | **T** | **-** | **T** | **A** | **T** | **T** | **T** | **A** | **C** | **-** | **C** | **T** | **C** | **T** | **-** | **A** | **T** | **C** | **G** | **C** | **C** | **T** | **A** | **G** | **G** | **A** | **G** | **C** | **A** | **C** | **-** | **C** | **G** | **C** | **T** | **T** |
|  | **4** | **T** | **T** | **T** | **A** | **T** | **C** | **T** | **G** | **C** | **-** | **C** | **T** | **C** | **T** | **A** | **A** | **T** | **C** | **G** | **T** | **G** | **T** | **A** | **G** | **A** | **G** | **A** | **C** | **A** | **T** | **-** | **C** | **G** | **C** | **T** | **T** |
|  | **1** | **T** | **-** | **T** | **A** | **T** | **T** | **T** | **G** | **C** | **-** | **C** | **T** | **C** | **T** | **-** | **A** | **T** | **C** | **G** | **C** | **C** | **T** | **A** | **G** | **G** | **A** | **G** | **C** | **A** | **C** | **-** | **C** | **G** | **C** | **T** | **T** |
|  | **1** | **T** | **T** | **T** | **A** | **T** | **T** | **T** | **A** | **C** | **-** | **C** | **T** | **C** | **T** | **-** | **A** | **T** | **C** | **G** | **T** | **G** | **T** | **A** | **G** | **A** | **G** | **A** | **C** | **A** | **T** | **-** | **C** | **G** | **C** | **T** | **T** |
|  | **2** | **T** | **T** | **T** | **A** | **T** | **C** | **T** | **G** | **C** | **-** | **C** | **T** | **C** | **T** | **A** | **A** | **T** | **C** | **G** | **T** | **G** | **T** | **A** | **G** | **G** | **A** | **G** | **C** | **A** | **C** | **-** | **C** | **G** | **C** | **T** | **T** |
|  |  |  |  |  |  |  |  |  |  |  |  |  |  |  |  |  |  |  |  |  |  |  |  |  |  |  |  |  |  |  |  |  |  |  |  |  |  |
| **DH147** | **2** | **T** | **-** | **T** | **A** | **T** | **T** | **T** | **A** | **C** | **-** | **C** | **T** | **C** | **T** | **-** | **A** | **T** | **C** | **G** | **C** | **C** | **T** | **A** | **G** | **G** | **A** | **G** | **C** | **A** | **C** | **-** | **C** | **G** | **C** | **T** | **T** |
|  | **4** | **T** | **T** | **T** | **A** | **T** | **C** | **T** | **G** | **C** | **-** | **C** | **T** | **C** | **T** | **A** | **A** | **T** | **C** | **G** | **T** | **G** | **T** | **A** | **G** | **A** | **A** | **A** | **C** | **A** | **T** | **-** | **C** | **G** | **C** | **T** | **T** |
|  | **1** | **T** | **-** | **T** | **A** | **T** | **T** | **T** | **A** | **C** | **-** | **C** | **T** | **C** | **T** | **A** | **A** | **T** | **C** | **G** | **T** | **G** | **T** | **A** | **G** | **A** | **G** | **A** | **C** | **A** | **T** | **-** | **C** | **G** | **C** | **T** | **T** |
|  | **1** | **T** | **-** | **T** | **A** | **T** | **C** | **T** | **G** | **C** | **-** | **C** | **T** | **C** | **T** | **A** | **A** | **T** | **C** | **G** | **T** | **G** | **T** | **A** | **G** | **G** | **A** | **G** | **C** | **A** | **C** | **-** | **C** | **G** | **C** | **T** | **T** |
|  | **1** | **T** | **T** | **T** | **A** | **T** | **C** | **T** | **G** | **C** | **-** | **C** | **T** | **C** | **T** | **A** | **A** | **T** | **C** | **G** | **T** | **G** | **T** | **A** | **G** | **G** | **A** | **G** | **C** | **A** | **C** | **-** | **C** | **G** | **C** | **T** | **T** |
|  |  |  |  |  |  |  |  |  |  |  |  |  |  |  |  |  |  |  |  |  |  |  |  |  |  |  |  |  |  |  |  |  |  |  |  |  |  |
| **DH16115** | **23** | **T** | **-** | **T** | **A** | **T** | **T** | **T** | **A** | **C** | **-** | **C** | **T** | **C** | **T** | **-** | **A** | **T** | **C** | **G** | **C** | **C** | **T** | **A** | **G** | **G** | **A** | **G** | **C** | **A** | **C** | **-** | **C** | **G** | **C** | **T** | **T** |
|  | **3** | **T** | **T** | **T** | **A** | **T** | **C** | **T** | **G** | **C** | **-** | **C** | **T** | **C** | **T** | **A** | **A** | **T** | **C** | **G** | **T** | **G** | **T** | **A** | **G** | **A** | **G** | **A** | **C** | **A** | **T** | **-** | **C** | **G** | **C** | **T** | **T** |
|  | **2** | **T** | **-** | **T** | **A** | **T** | **T** | **T** | **A** | **C** | **-** | **C** | **T** | **C** | **T** | **-** | **A** | **T** | **C** | **G** | **T** | **G** | **T** | **A** | **G** | **G** | **A** | **G** | **C** | **A** | **T** | **-** | **C** | **G** | **C** | **T** | **T** |
|  | **1** | **T** | **-** | **T** | **A** | **T** | **T** | **T** | **A** | **C** | **-** | **C** | **T** | **C** | **T** | **-** | **A** | **T** | **C** | **G** | **C** | **C** | **T** | **A** | **G** | **A** | **A** | **G** | **C** | **A** | **C** | **-** | **C** | **G** | **C** | **T** | **T** |
|  | **2** | **T** | **-** | **T** | **A** | **T** | **T** | **T** | **A** | **C** | **-** | **C** | **T** | **C** | **T** | **-** | **A** | **T** | **C** | **G** | **T** | **G** | **T** | **A** | **G** | **G** | **A** | **G** | **C** | **A** | **C** | **-** | **C** | **G** | **C** | **T** | **T** |
|  | **3** | **T** | **-** | **T** | **A** | **T** | **T** | **T** | **A** | **C** | **-** | **C** | **T** | **C** | **T** | **-** | **A** | **T** | **C** | **G** | **T** | **G** | **T** | **A** | **G** | **A** | **G** | **A** | **C** | **A** | **T** | **-** | **C** | **G** | **C** | **T** | **T** |
|  | **1** | **T** | **-** | **T** | **A** | **T** | **T** | **T** | **A** | **C** | **-** | **C** | **T** | **C** | **T** | **-** | **A** | **T** | **C** | **G** | **C** | **C** | **T** | **A** | **G** | **A** | **G** | **A** | **C** | **A** | **T** | **-** | **C** | **G** | **C** | **T** | **T** |
|  | **1** | **T** | **-** | **T** | **A** | **T** | **T** | **T** | **A** | **C** | **-** | **C** | **T** | **C** | **T** | **A** | **A** | **T** | **C** | **G** | **T** | **G** | **T** | **A** | **G** | **G** | **A** | **G** | **C** | **A** | **C** | **-** | **C** | **G** | **C** | **T** | **T** |
|  | **4** | **T** | **-** | **T** | **A** | **T** | **T** | **T** | **A** | **C** | **-** | **C** | **T** | **C** | **T** | **A** | **A** | **T** | **C** | **G** | **C** | **C** | **T** | **A** | **G** | **G** | **A** | **G** | **C** | **A** | **C** | **-** | **C** | **G** | **C** | **T** | **T** |
|  | **3** | **T** | **-** | **T** | **A** | **T** | **C** | **T** | **G** | **C** | **-** | **C** | **T** | **C** | **T** | **A** | **A** | **T** | **C** | **G** | **T** | **G** | **T** | **A** | **G** | **A** | **G** | **A** | **C** | **A** | **T** | **-** | **C** | **G** | **C** | **T** | **T** |
|  | **1** | **T** | **-** | **T** | **A** | **T** | **C** | **T** | **G** | **C** | **-** | **C** | **T** | **C** | **T** | **A** | **A** | **T** | **C** | **G** | **T** | **G** | **T** | **A** | **G** | **G** | **G** | **A** | **C** | **A** | **T** | **-** | **C** | **G** | **C** | **T** | **T** |
|  | **1** | **T** | **T** | **T** | **A** | **T** | **C** | **T** | **G** | **C** | **-** | **C** | **T** | **C** | **T** | **-** | **A** | **T** | **C** | **G** | **C** | **C** | **T** | **A** | **G** | **G** | **A** | **G** | **C** | **A** | **C** | **-** | **C** | **G** | **C** | **T** | **T** |
|  | **1** | **T** | **T** | **T** | **A** | **T** | **C** | **T** | **G** | **C** | **-** | **C** | **T** | **C** | **T** | **-** | **A** | **T** | **C** | **G** | **C** | **C** | **T** | **A** | **G** | **G** | **A** | **G** | **C** | **A** | **T** | **-** | **C** | **G** | **C** | **T** | **T** |
|  | **2** | **T** | **T** | **T** | **A** | **T** | **C** | **T** | **G** | **C** | **-** | **C** | **T** | **C** | **T** | **-** | **A** | **T** | **C** | **G** | **T** | **G** | **T** | **A** | **G** | **G** | **A** | **G** | **C** | **A** | **C** | **-** | **C** | **G** | **C** | **T** | **T** |
|  | **3** | **T** | **T** | **T** | **A** | **T** | **C** | **T** | **G** | **C** | **-** | **C** | **T** | **C** | **T** | **-** | **A** | **T** | **C** | **G** | **C** | **C** | **T** | **A** | **G** | **G** | **A** | **G** | **C** | **A** | **C** | **-** | **C** | **G** | **C** | **T** | **T** |
|  | **1** | **T** | **T** | **T** | **A** | **T** | **C** | **T** | **G** | **C** | **-** | **C** | **T** | **C** | **T** | **-** | **A** | **T** | **C** | **G** | **C** | **C** | **T** | **A** | **G** | **A** | **G** | **A** | **C** | **A** | **T** | **-** | **C** | **G** | **C** | **T** | **T** |
|  | **1** | **T** | **T** | **T** | **A** | **T** | **T** | **T** | **A** | **C** | **-** | **C** | **T** | **C** | **T** | **A** | **A** | **T** | **C** | **G** | **T** | **G** | **T** | **A** | **G** | **G** | **A** | **G** | **C** | **A** | **C** | **-** | **C** | **G** | **C** | **T** | **T** |
|  | **1** | **T** | **T** | **T** | **A** | **T** | **C** | **T** | **G** | **C** | **-** | **C** | **T** | **C** | **T** | **A** | **A** | **T** | **C** | **G** | **C** | **C** | **T** | **A** | **G** | **G** | **A** | **G** | **C** | **A** | **C** | **-** | **C** | **G** | **C** | **T** | **T** |
|  | **2** | **T** | **T** | **T** | **A** | **T** | **C** | **T** | **G** | **C** | **-** | **C** | **T** | **C** | **T** | **A** | **A** | **T** | **C** | **G** | **T** | **G** | **T** | **A** | **G** | **G** | **A** | **G** | **C** | **A** | **C** | **-** | **C** | **G** | **C** | **T** | **T** |
|  | **1** | **T** | **T** | **T** | **A** | **T** | **C** | **T** | **G** | **C** | **-** | **C** | **T** | **C** | **T** | **A** | **A** | **T** | **C** | **G** | **T** | **G** | **T** | **A** | **G** | **A** | **G** | **G** | **C** | **A** | **C** | **-** | **C** | **G** | **C** | **T** | **T** |
|  | **1** | **T** | **T** | **T** | **A** | **T** | **C** | **T** | **G** | **C** | **-** | **C** | **T** | **C** | **T** | **A** | **A** | **T** | **C** | **G** | **T** | **G** | **T** | **A** | **G** | **A** | **G** | **A** | **C** | **A** | **T** | **-** | **C** | **G** | **C** | **T** | **T** |
|  | **1** | **T** | **T** | **T** | **A** | **T** | **C** | **T** | **G** | **C** | **-** | **C** | **T** | **C** | **T** | **A** | **A** | **T** | **C** | **G** | **C** | **C** | **T** | **A** | **G** | **A** | **G** | **A** | **C** | **A** | **T** | **-** | **C** | **G** | **C** | **T** | **T** |
|  | **1** | **T** | **T** | **T** | **A** | **T** | **C** | **T** | **G** | **C** | **-** | **C** | **T** | **C** | **T** | **A** | **A** | **T** | **C** | **G** | **T** | **G** | **T** | **A** | **G** | **A** | **G** | **A** | **C** | **A** | **C** | **-** | **C** | **G** | **C** | **T** | **T** |

^1^ number of clones with the corresponding profile
